# Phosphogypsum-Based Composite Cementitious Porous Filter Media: Performance Regulation and Phosphate Adsorption Mechanism

**DOI:** 10.3390/polym18202487

**Published:** 2026-10-12

**Authors:** Yu Xia, Jianjun Xia, Xishu Xu, Dianxi Yu, Zhiheng Zhang, Ting Xia

**Affiliations:** 1 College of Urban Construction, Nanjing Tech University, Puzhu Road 30, Nanjing 211816, China; yxx570@gmail.com (Y.X.); xiajianjun2022@163.com (J.X.); 13809087108@163.com (X.X.); 13770616672@163.com (D.Y.); 15950596724@163.com (Z.Z.); 2 College of Engineering, Carnegie Mellon University, Forbes Ave, 119 Porter Hall, 5000, Pittsburgh, PA 15213, USA

**Keywords:** phosphogypsum, fly ash, redispersible polymer powder, porous filter media, alkali activation, phosphate removal, solid waste resource utilization

## Abstract

**Highlights:**

• Alkali-activated phosphogypsum-based porous filter media wereprepared via animal protein foaming and RPP toughening modification.
• The synergistic effect of calcium-induced precipitation and mesoporous physical adsorption dominates the phosphorus removal performance.
• The obtained filter media present low hazardous ion leaching performance and good environmental safety for water treatment.
• This preparation route achieves efficient resource utilization of waste phosphogypsum and eutrophic water purification.

**Abstract:**

In this work, a high-dosage phosphogypsum (PG) composite cementitious system was fabricated via alkali activation, using 50 wt.% PG, 15 wt.% fly ash and 35 wt.% ordinary Portland cement. Redispersible polymer powder (RPP) was incorporated to improve matrix toughness, and animal protein foaming was employed to construct continuous mesoporous structures for water treatment. Key preparation parameters were optimized through single-factor experiments. The optimized porous filter media present a bulk density of 0.809 g/cm^3^, a 1 h water absorption rate of 39%, and a breakage and wear rate of 4.89%. In static adsorption tests with 40 mg/L simulated phosphate wastewater, the filter media achieve a phosphate-removal rate of 71.81% within 6 h, which is significantly superior to that of commercial sintered ceramsite (50.28%). Combined characterization using BET, SEM, XRD, XRF and FT-IR demonstrates that phosphate removal mainly relies on calcium-induced chemical precipitation, with mesoporous physical adsorption playing an auxiliary role. Leaching tests verify that all heavy metal leaching concentrations of the prepared filter media satisfy the GB 5085.3-2007 standard, while fluoride release can be detected under strong acid conditions. This polymer modification strategy facilitates large-scale PG solid waste disposal and eutrophic water remediation, providing a practical route for preparing low-cost polymer-inorganic porous filter media for water purification.

## 1. Introduction

With the continuous expansion of the wet-process phosphoric acid industry, 4–5 tons of by-product phosphogypsum (PG) are generated per ton of phosphoric acid produced. The global cumulative stockpiles of PG in open-air dumps have exceeded 6 billion tons, with an annual output of approximately 280 million tons. In China, 80 million tons of PG are produced annually, making it the world’s largest PG-producing country [1]. Owing to difficulties in its utilization, long-term stockpiling of PG occupies large areas of land. PG contains soluble impurities, including phosphorus, fluorine, radium, and cadmium, which can leach out due to rainfall, triggering combined contamination of soil and groundwater and posing persistent ecological safety risks. Building-material utilization represents the dominant disposal route for PG; nevertheless, its comprehensive utilization rate remains low. Therefore, it is urgently needed to develop novel resource-recovery pathways featuring high added value and large-scale disposal capacity [2].

In terms of material properties, PG is mainly composed of calcium sulfate dihydrate. After heat treatment, PG transforms into hemihydrate and anhydrous PG with cementitious activity. Meanwhile, its matrix is rich in free Ca^2+^ and SO_4_^2−^. These ions can immobilize oxo-anions and diverse metal ions in wastewater via ion-exchange and precipitation reactions, making PG with inherent potential for purifying wastewater. Nevertheless, existing studies mostly develop PG merely as civil-engineering substrates; integrated composite systems that simultaneously realize building-material application and water-treatment functions are severely lacking.

Raw PG exhibits extremely low hydraulic activity, and its hydration and mechanical properties need to be regulated via calcination, mechanical activation, solid-waste compounding, alkali-activation, polymer doping and other approaches [3]. Qu et al. [4] systematically reviewed studies on PG-based cementitious materials published between 2014 and 2024 and concluded that systems with high PG content often have several key drawbacks, including inadequate hydration, later-age strength degradation and facile impurity leaching. Therefore, modification methods such as alkali activation and composite blending with other materials are required. Shi et al. [5] made composite samples with 50.57 wt.% PG mixed with slag, fly ash (FA) and slaked lime, which reached a 28-day compressive strength of 23.57 MPa. However, high PG contents decrease the alkalinity of the system, resulting in fewer hydration products forming, and the matrix becomes porous. Tang et al. [6] and Cao et al. [7] established several ternary and quaternary collaborative solid-waste systems in their separate studies. Their findings proved that the mixture of PG, slag and carbide slag yielded optimal hydration performance. Ettringite (denoted as AFt, chemical formula 3CaO·Al_2_O_3_·3CaSO_4_·32H_2_O) and C-(A)-S-H gel, namely calcium-(aluminum)-silicate hydrate gel, an amorphous hydration product with variable composition where partial Al substitutes Si in the silicate tetrahedral network, formed an interlocked skeleton within the system. Heavy-metal, phosphorus and fluorine impurities could be stably immobilized via coprecipitation and lattice encapsulation. Lin et al. [8] and Fu et al. [9] studied how inorganic additives, including silica fume and mineral admixtures, affect the drying shrinkage and durability of substrates. Silica fume could increase the early-stage hydration rate of cementitious systems, but its high raw-material cost limits its large-scale practical application. For polymer-modified systems, Chen et al. [10] carried out multi-scale modification by using styrene-butadiene rubber (SBR)/poly(vinyl alcohol) (PVA) polymers together with mineral fibers. Their experimental results indicated that polymers could form fibrous networks inside pores to disperse stress and improve strength. Even so, their system was only developed for dense building mortar. Sina et al. [11] adjusted material strength by using bio-based microcrystalline cellulose. Similarly, their work was limited to non-porous dense pastes, and they did not design porous structures for water-treatment purposes.

The abundant active calcium-binding sites within the PG matrix render them promising candidates for wastewater treatment, with existing PG-based water purification applications primarily divided into powder adsorption and molded granular filtration media [12]. Although the crystalline La-Al co-loaded Fe_3_O_4_ magnetic adsorbent achieved a high maximum phosphate adsorption capacity of 94.3 mg/g and maintained 89.08% of its initial performance after five adsorption–desorption cycles, it was fabricated using rare-earth-containing chemical reagents rather than reusing bulk industrial solid waste resources [13]. Yan et al. [14] and Li et al. [15] prepared different PG-based powder adsorbents. Their test results showed that raw and modified PG powders have excellent adsorption capacities for lead, arsenic, and organic dyes. Nevertheless, direct dosing of these fine powders may block pipelines, and heavy-metal impurities may leach out again, bringing secondary pollution risks.

To address the practical limitations of powder-type PG adsorbents, multiple studies have explored granulation and forming techniques for PG-based materials. Gan et al. [16] and Huang et al. [17] produced PG ceramsite by high-temperature calcination at 900–1000 °C. High-temperature treatment provides stable calcium sources for phosphate precipitation, whereas intensive energy consumption substantially increases operational costs and restricts large-scale application. Ding et al. [18] used a disc-type cold-bond granulation process at ambient temperature to fabricate lightweight aggregates with 60–80 wt.% PG, in which AFt and C-S-H gel formed during hydration could coat and immobilize heavy-metal and soluble-phosphorus impurities, yet the granular skeleton is susceptible to hydraulic erosion under long-term flowing water conditions, limiting its application primarily to concrete filling rather than water treatment.

Currently, various lightweight pore-forming techniques have been developed for PG-based materials, such as inorganic microsphere filling, spontaneous foaming using hydrogen peroxide or microorganisms, SAP internal-template granulation, polyurethane core-material coating, and calcined PG coated with super-absorbent polymers [19,20,21]. However, existing lightweight cold-granulated materials with PG as the main component are mainly designed for building thermal-insulation walls. Their pore structures are optimized for thermal insulation performance, and most pores exist as isolated closed pores. They lack continuous interconnected mesoporous channels, which are required for sufficient contact between pollutants and active calcium sites. Therefore, these materials struggle to meet the mass-transfer requirements of dynamic-water-treatment filter media.

A comprehensive literature review indicates that current PG-based water treatment materials still present three key limitations, which are closely associated with polymer regulation and porous structure design. First, alkali-activated cementitious systems with high PG content exhibit brittle inorganic hydration matrices and severe drying shrinkage cracking, while the microscale mechanisms of organic–inorganic composite toughening are still poorly clarified. Second, existing PG-based adsorbents for phosphorus removal are mainly limited to powders and high-temperature sintered ceramsite, and lightweight cold granulation techniques have rarely been adopted for such materials. Third, most current studies separate the architectural and water-treatment application scenarios, and no integrated forming system is available to simultaneously realize PG solid waste disposal and eutrophic wastewater remediation.

Accordingly, this study develops a high-dose PG composite system activated by ordinary Portland cement, FA, and water glass. Redispersible polymer powder (RPP) is introduced to enhance matrix toughness and suppress cracking, while controllable animal protein foaming is adopted to construct continuous mesoporous structures. The resulting polymer-modified porous PG filter media can sustainably release calcium ions to immobilize phosphate via chemical precipitation, achieving dual functions of building thermal insulation and efficient water purification. This work systematically investigates the synergistic hydration behavior of organic–inorganic composite phases and the mesoporous structure regulation mechanism. Static phosphorus adsorption experiments and leaching toxicity tests are conducted to evaluate pollutant removal performance and environmental safety. This study provides feasible technical support for the development of low-cost, multifunctional solid-waste-based filter materials for practical water treatment.

## 2. Experimental Materials and Methods

### 2.1. Raw Materials

#### 2.1.1. Phosphogypsum (PG)

PG was obtained from an industrial PG stockpile in Deyang City, Sichuan Province, China. It is a typical by-product of wet-process phosphoric acid production, and the sample is long-term aged PG. The raw material contains large amounts of calcium sulfate dihydrate, as well as impurities including free water, soluble phosphate and soluble fluoride. This PG sample has a loss on ignition (LOI) of 20.18% at 850 °C. Prior to experimentation, raw PG was dried at 105 °C for 2 h to remove surface-free moisture. Drying eliminates free water to restrain the migration of soluble salts and reduce surface-adsorbed soluble impurities, while it cannot remove soluble phosphate and fluoride; these soluble impurities are immobilized via hydration and coprecipitation inside the alkali-activated matrix during curing. It was then ground using a ball mill for 45 min, yielding gray powder with a particle size below 75 μm. Its chemical composition is presented in Table 1.

#### 2.1.2. Supplementary Cementitious Materials

Ordinary Portland Cement (OPC, 42.5 grade, with a standard 28-day compressive strength ≥ 42.5 MPa): Commercially available from Nanjing Zhonglian Cement Co., Ltd., Nanjing, China. It drives hydration reactions to form hydraulic gel phases, binds PG particles together, and enhances the overall cohesion and durability of the PG-based cementitious system.

Fly ash (FA): Grade-I fly ash obtained from Shazhou Power Plant, Zhangjiagang, China. Its chemical composition is listed in Table 1. The amorphous SiO_2_ and Al_2_O_3_ in FA are alkali-active components, which can be depolymerized and re-polymerized under an alkaline environment to form Si–OH and Al–OH group complexes, further generating secondary hydration gels. Appropriate incorporation of FA effectively reduces OPC consumption while guaranteeing the long-term mechanical strength of PG-based composites.

Alkali activator: Industrial sodium silicate with a modulus of 3.0, supplied by J&K Scientific Ltd., Shanghai, China. It provides sufficient OH^−^ to destroy the stable glass-phase structure of fly ash, thereby stimulating the pozzolanic activity of raw materials. Meanwhile, it serves as an exogenous silicon source to supplement the formation of hydrated gel minerals.

Redispersible polymer powder (RPP): A commercially available ethylene-vinyl acetate (VAE)-based powder supplied by Anhui Wanwei Group Co., Ltd., Chaohu, China. During hydration and hardening, RPP forms continuous flexible polymer films within the matrix, which benefits the improvement of toughness, flexural strength and water stability of rigid PG–cement composites.

Animal protein foaming agent: A commercial animal-protein foaming agent with high foam stability, low bleeding and settlement loss, supplied by Jiangsu Baihehe New Materials Co., Ltd., Suzhou, China. It functions to promote the formation of porous channel structures within the cementitious system. It was screened by comparison with amino sulfonate, plant protein, and composite foaming agents in this experiment. The plant protein foaming agent was also obtained from Jiangsu Baihehe New Materials Co., Ltd., Suzhou, China, while the amino sulfonate foaming agent was purchased from New Equipment Co., Ltd., Zhengzhou, China.

#### 2.1.3. Reagents and Comparison Filter Media

Analytical reagents: NH_4_Cl, potassium dichromate, NaOH, potassium sodium tartrate, potassium dihydrogen phosphate, and ascorbic acid were purchased from Wanqing Chemical Glass Instrument Co., Ltd., Nanjing, China. These reagents were applied for total phosphorus (TP) detection using the ammonium molybdate spectrophotometric method in water sample analysis.

Control filler: Commercially available sintered clay ceramsite (specific surface area 8.75 m^2^/g, apparent density 1.2 g/cm^3^, particle size of 10–20 mm), purchased from Xuyi Boshi Environmental Protection Materials Co., Ltd., Xuyi, Jiangsu, China, which was used for parallel phosphorus-removal comparison tests.

### 2.2. Experimental Apparatus

The instruments and equipment used in the experiments are listed in Table 2.

### 2.3. Specimen Preparation

**Figure 1:**
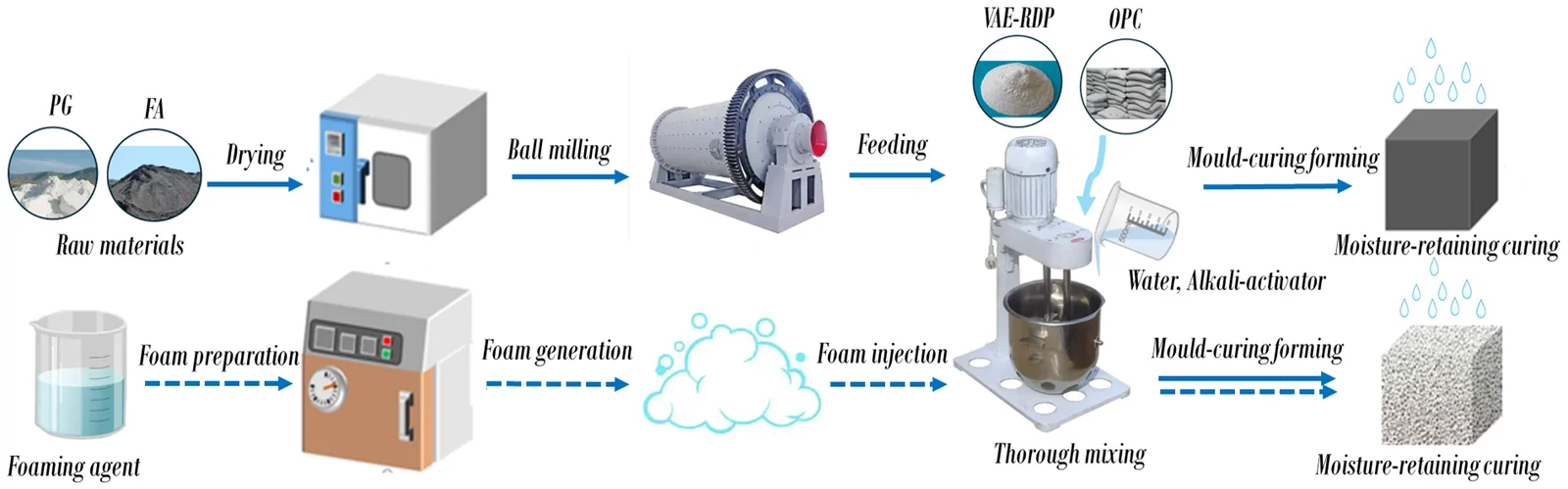
Preparation flow chart of PG-based composite cementitious materials and porous filter media. Note: Solid-line flows represent the preparation process of PG-based composite cementitious materials, while flows added with dashed lines denote the preparation procedure of PG-based filter media.

The preparation process consisted of two stages: (i) forming of PG-based composite cementitious matrix; and (ii) secondary preparation of foamed porous filter media. The preparation flow chart is shown in Figure 1.

#### 2.3.1. Preparation of PG-Based Composite Cementitious Matrix Specimens

In the fixed cementitious system, PG was maintained at 50 wt.%, and cement was partially replaced by FA at equal mass. The experimental variables included a water-to-binder ratio of 0.2–0.6, FA replacement content of 0–30 wt.%, alkali activator dosage of 0–10 wt.%, and RPP dosage of 0–2.5 wt.%.

Mixing steps were as follows: Dried and ball-milled PG, OPC and FA powders were first dry-mixed for 1 min. Predetermined amounts of water, alkali activator and RPP were then added, and the mixture was stirred at a constant speed of 400 rpm for 2 min to obtain a homogeneous paste. The paste was poured into standard triple-gang molds of 40 mm × 40 mm × 160 mm. Specimens were kept at room temperature for 24 h prior to demolding and subsequently placed in a standard curing chamber for 3 d, 14 d and 28 d for subsequent strength and setting-time tests.

#### 2.3.2. Preparation of Porous PG Filter Media

Foaming pore-forming experiments were carried out based on the optimal mix proportion obtained in Section 2.3.1. The animal-protein foaming agent described in Section 2.1.2 was adopted. The foaming agent was first diluted with deionized water according to the recommended dilution ratio to produce stable foam using a foaming machine. Tests were performed at foam volume fractions of 2%, 4%, 6%, 8%, and 10% relative to the total mass of cementitious powders, with a mixing-time gradient of 0.5–3 min and a constant stirring speed of 400 rpm. Pre-prepared foam was continuously introduced into the cementitious paste, followed by low-speed mixing for 30–90 s to achieve uniform foam dispersion. The mixture was cast into 15 mm × 15 mm × 15 mm molds, and after static storage at room temperature for 24 h, specimens were demolded and standard-cured for 28 d prior to pore-structure, adsorption and leaching tests.

### 2.4. Testing and Calculation Methods

#### 2.4.1. Macro-Property Tests of Matrix Specimens

Setting time was measured by a Vicat apparatus in accordance with GB/T 1346-2024, Methods for testing water consumption of standard consistency, setting time and soundness of cement. The initial setting time was defined when the test needle penetrated the paste to 4 mm ± 1 mm above the bottom plate, and the final setting time was reached when no obvious indentation was left by the annular attachment.

Compressive and flexural strengths were tested following GB/T 9776-2022, Building gypsum, at loading rates of 1000 N/s for the compressive test and 50 N/s for the flexural test. The average value was calculated from three parallel specimens for each group.

The compressive strength was calculated by Equation (1):

(1) $$F_{c}=P/A$$

where *F_c_* is the compressive strength (MPa); *P* is the maximum load (N); and *A* is the loaded area (mm^2^).

The flexural strength was calculated by Equation (2):

(2) $$f_{r}=3FL/\left(2bh^{2}\right)$$

where *f_r_* is the flexural strength (MPa); *F* is the fracture load (N); *L* is the support span (mm); and *b* and *h* are the width and height of the specimen cross-section (mm).

#### 2.4.2. Foam Performance Evaluation

After the foaming solution was prepared, it was pumped into a foaming machine. The generated foam was collected in a 3000 mL graduated cylinder to determine the foam expansion ratio, 1 h sedimentation rate, and 1 h bleeding rate.

The foam expansion ratio was calculated using Equation (3):

(3) $$N=V_{w}/V_{q}$$

where *N* is the foam expansion ratio; *V_w_* is the total foam volume after foaming (mL); and *V_q_* is the volume of the original foaming solution (mL).

The 1 h foam sedimentation rate was calculated using Equation (4):

(4) $$S=\left(V_{w}-V_{h}\right)/V_{w}\times 100\%$$

where *S* is the 1 h foam sedimentation rate (%); and *V_h_* is the total volume of foam and bleeding water after 1 h of static placement (mL).

The 1 h bleeding rate was calculated using Equation (5):

(5) $$B=M_{2}/\left(M_{1}-M_{0}\right)\times 100\%$$

where *B* is the 1 h foam bleeding rate (%); *M*_0_ is the mass of the empty graduated cylinder (g); *M*_1_ is the total mass of the cylinder and fresh foam (g); and *M*_2_ is the mass of bleeding water separated after 1 h of standing (g).

#### 2.4.3. Physical Properties of Porous Filter

The bulk density, 1 h water absorption, and sum of breakage and wear rates were tested in accordance with GB/T 17431.2-2010 (Lightweight aggregates and their test methods——Part 2: Test methods for lightweight aggregates) and CJ/T 299-2008 (Artificial ceramsite filter media for water treatment).

The bulk density was calculated by Equation (6):

(6) ρ=G/V

where *ρ* is the bulk density (g/cm^3^); *G* is the average mass of specimens dried to constant weight at 110 °C (g); and *V* is the geometric volume of the specimen (cm^3^).

The 1 h water absorption was calculated by Equation (7):

(7) $$W=\left(M_{4}-M_{3}\right)/M_{3}\times 100\%$$

where *W* is the 1 h water absorption rate (%); *M*_3_ is the mass of dry filter specimens (g); and *M*_4_ is the specimen mass after 1 h water immersion and surface drying (g).

The sum of breakage and abrasion rates was determined by placing 100 g of dry filter material sieved through a 0.5 mm screen together with six Φ8 mm steel balls in a sealed container for 15 min of vibration at 150 times per minute. The mass of fine particles passing through the 0.5 mm sieve after vibration was weighed for calculation:

(8) $$C=M_{6}/M_{5}\times 100\%$$

where C is the sum of breakage and wear rate (%); *M*_5_ is the initial mass of filter material (g); and *M*_6_ is the mass of fine powder passing through the 0.5 mm sieve after vibration (g).

#### 2.4.4. Static Phosphate Adsorption Test

Simulated phosphate wastewater with an initial TP concentration of 40 mg/L was prepared with potassium dihydrogen phosphate. A volume of 100 mL of the prepared solution was poured into a 250 mL conical flask, and 5 g of self-made PG filter media and sintered ceramsite (particle size of 10–20 mm) were added separately. The samples were shaken continuously in a thermostatic shaker at 25 °C and collected at 1, 2, 3, 4, 5, and 6 h. After centrifugation at 2000 rpm for 30 min, the supernatant was diluted, and the residual total phosphorus concentration was measured via the ammonium molybdate spectrophotometric method.

Phosphate-removal efficiency was calculated by Equation (9):

(9) $$R=\left(C_{0}-C_{t}\right)/C_{0}\times 100\%$$

where *R* is the phosphate-removal efficiency (%); *C*_0_ is the initial TP concentration (mg/L); and *C_t_* is the residual TP concentration at time *t* (mg/L).

#### 2.4.5. Material Leaching Test

Leaching tests for the filter media were conducted in accordance with GB 5085.3-2007, Identification standards for hazardous wastes—identification for extraction toxicity. Cu, Zn, Cd, Pb, Cr, Hg, As and fluoride were measured to evaluate the environmental safety of the filter media in water-treatment applications.

#### 2.4.6. Multi-Scale Material Characterization

Microscopic characterizations were carried out to investigate the filter media samples. SEM observation was performed at an accelerating voltage of 3.00 kV with a magnification of 5000 to compare surface pores and crystal morphologies before and after adsorption. XRD tests used a Cu target over the scanning range of 5–60° to analyze hydration products and newly formed precipitated phases after adsorption. FT-IR spectra were collected from 4000 to 400 cm^−1^ to identify changes in functional groups, including Si-O, –OH and C=O. Nitrogen adsorption–desorption measurements (BET) were conducted at 77 K to obtain the specific surface area, pore volume, average pore size and mesopore-size distribution. XRF was applied to determine the bulk elemental composition of the filter media.

#### 2.4.7. Data Processing and Statistical Analysis

Experimental data were statistically processed using IBM SPSS Statistics 26. All figures were plotted with Origin 2025.

## 3. Results and Analysis

### 3.1. Single-Factor Regulation on Performance Evolution of PG Composite Cementitious Matrix

#### 3.1.1. Effect of Water–Binder Ratio on Setting Time and Mechanical Strength

**Figure 2:**
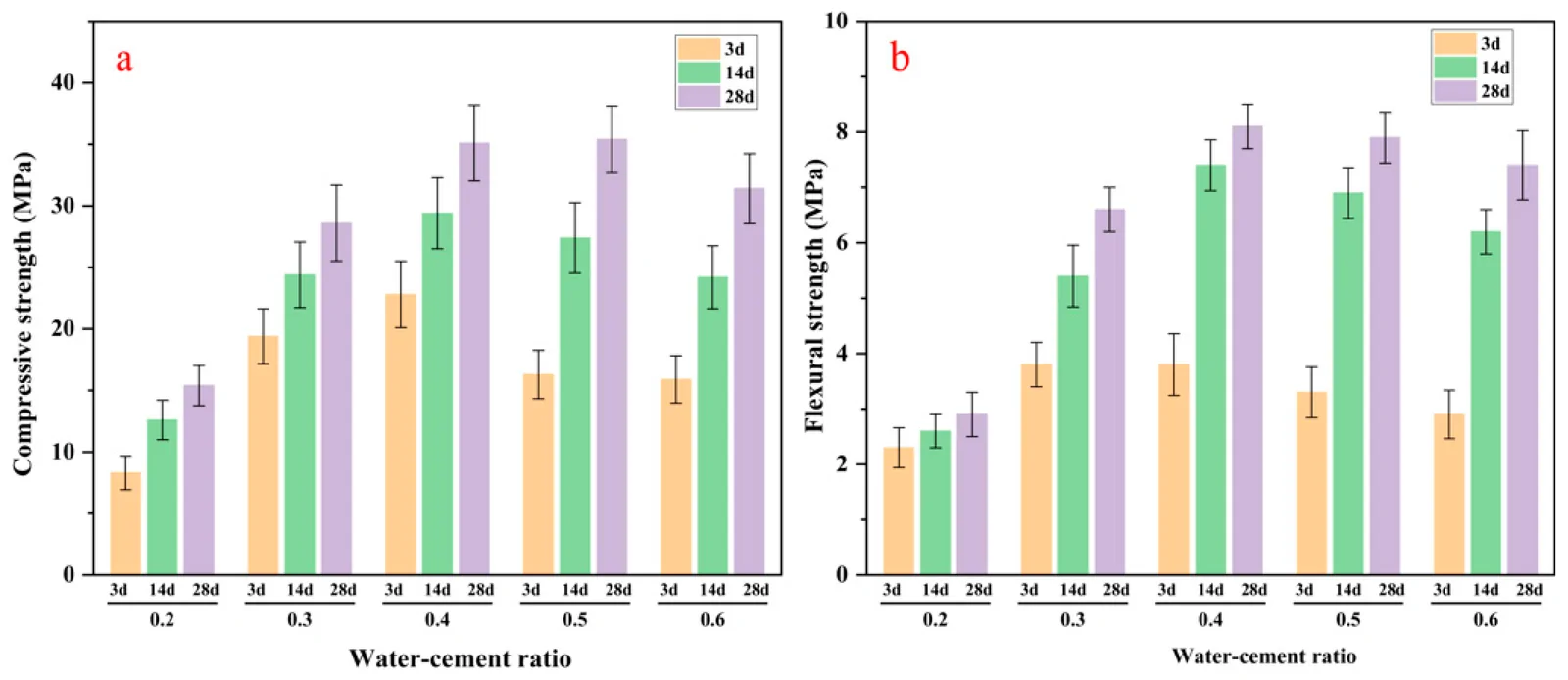
Effects of water–binder ratio on mechanical strengths of cementitious materials: (**a**) compressive strength; (**b**) flexural strength.

The water–binder ratio is a critical parameter governing the hydration degree, workability and mechanical properties of cementitious paste, and it greatly influences internal pore evolution and hydration reactions within the matrix system. In this experiment, the dosages of PG and OPC were both fixed at 500 g, with an additional 4% alkali activator and 1% RPP. Five water–binder ratios (0.2, 0.3, 0.4, 0.5, and 0.6) were selected for testing. The influences of varying water–binder ratios on the properties of cementitious materials are presented in Table 3 and Figure 2.

The setting time results indicate that both the initial and final setting times gradually prolonged with the increase in the water–binder ratio. At a water–binder ratio of 0.2, the initial and final setting times were 1.62 h and 7.20 h, respectively. Insufficient free water at this ratio promoted the rapid formation of dense C–S–H layers on the particle surface during the early hydration stage. Such dense hydration layers hindered water penetration into the interior of particles, retarded subsequent hydration reactions, and thus prolonged the overall setting process [22]. When the water–binder ratio increased to 0.3, sufficient water supply improved the fluidity of the paste and enabled fuller contact between mineral particles and water, reducing the initial setting time to 1.35 h. Further increases in the water–binder ratio introduced excessive free water, which diluted the ion concentration in the paste and slightly slowed down the hydration rate. Consequently, both initial and final setting times increased steadily [23]. At the maximum water–binder ratio of 0.6, the initial and final setting times reached 2.36 h and 8.12 h, respectively.

The compressive and flexural strengths at 3 d, 14 d and 28 d exhibited a single-peak trend, first increasing and then decreasing with the rise in water–binder ratio. Taking the 28 d compressive strength as an example, the value was only 15.6 MPa at a water–binder ratio of 0.2, reached a peak of 35.4 MPa at 0.4, and subsequently decreased to 31.4 MPa when the ratio increased to 0.6. At low water–binder ratios, insufficient free water restricted the hydration reaction, resulting in low production of cementitious products such as calcium silicate hydrate and AFt. The weak bonding between particles accordingly reduced the overall mechanical strength. A moderate increase in the water–binder ratio enabled sufficient hydration of mineral components. Abundant C-S-H gels and needle-like AFt filled internal pores and formed a dense microstructure, which effectively improved the compactness and mechanical performance of the matrix. However, an excessively high water–binder ratio left a large number of interconnected capillary pores inside the hardened paste. The increased porosity and stress concentration points further led to the degradation of late-stage strength [24]. Considering both the construction operable time and long-term mechanical properties, the optimal water–binder ratio was determined to be 0.4.

#### 3.1.2. Effect of FA Replacement of OPC on System Performance

In this test, the total dosage of PG was kept at 500 g. FA was used to replace OPC at equal mass, with seven replacement levels ranging from 0% to 30%. The water–binder ratio was fixed at 0.4, alkali activator at 6%, and RPP at 1%, to investigate how FA content influences setting behavior and mechanical strength.

**Figure 3:**
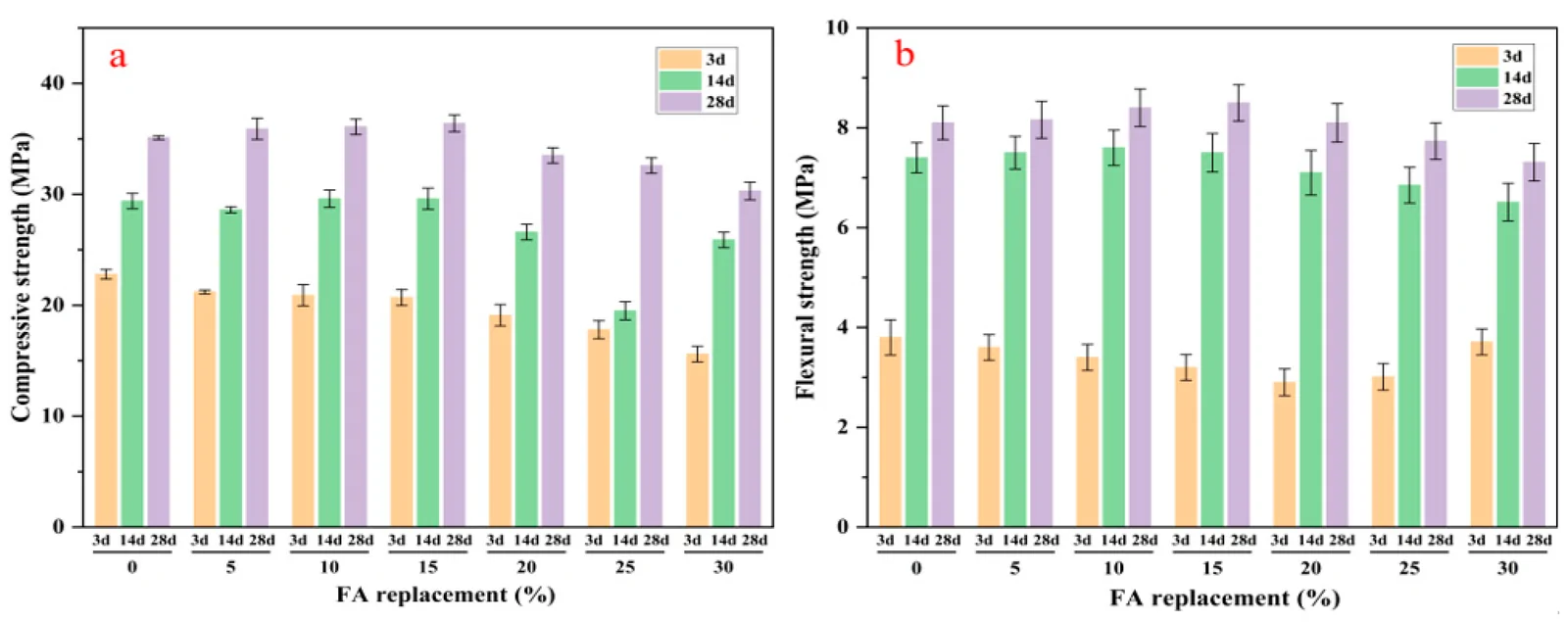
Effects of FA replacement of OPC on mechanical strengths of cementitious materials: (**a**) compressive strength; (**b**) flexural strength.

Test results show that both initial and final setting times increase with increasing FA content (Table 4 and Figure 3). At 30% FA replacement, the initial setting time reached 3.92 h, nearly 2.9 times longer than the group without FA. FA has a large specific surface area; its particle pores adsorb free water in the paste and reduce contact between OPC minerals and water, thus retarding early-age OPC hydration. Additionally, the aluminosilicate glass phase in FA exhibits low hydraulic reactivity, and its secondary hydration proceeds much more slowly than OPC clinker hydration. Combined, these two factors greatly prolong the setting time [25].

The mechanical results reveal that FA obviously reduces the early-age compressive strength, yet appropriate FA addition improves later-age strength through a pozzolanic reaction. When FA replacement stays below 15%, sufficient Ca(OH)_2_ from OPC hydration continuously activates amorphous SiO_2_ and Al_2_O_3_ in FA to trigger secondary hydration. Low calcium C A S H gel and AFt form and fill internal pores, compensating early strength loss and leading to a slight increase in 28 d strength. Once FA replacement exceeds 15%, the supply of Ca(OH)_2_ from hydration becomes inadequate. Large amounts of FA cannot fully take part in pozzolanic reactions, the fraction of inert powder rises, and the skeleton strength of the matrix keeps decreasing [26]. Flexural performance shows different trends. FA optimizes particle packing of the matrix. A small FA dosage refines internal pores and improves material toughness, so early-age flexural strength presents a trend of first decreasing and then increasing. Nevertheless, the benefit brought by particle size gradation fades gradually after long-term hydration. Considering setting time, as well as the balance between early and later-age mechanical performance, the optimal FA replacement ratio is determined as 15%, corresponding to 35% OPC dosage.

#### 3.1.3. Regulation of Alkali-Activator Dosage on the Performance of Cementitious System

**Figure 4:**
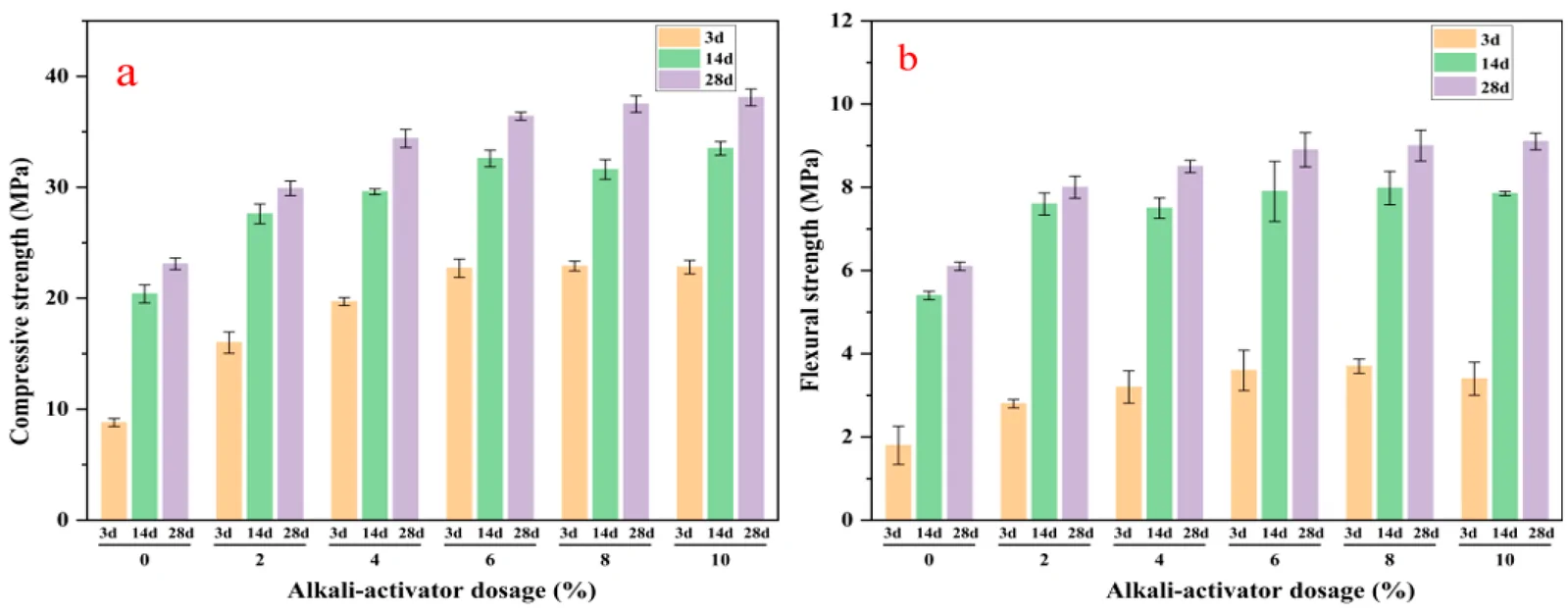
Effects of alkali-activator dosage on mechanical strengths of cementitious materials: (**a**) compressive strength; (**b**) flexural strength.

Based on the optimal proportion of PG, OPC and FA, six alkali-activator dosage levels ranging from 0% to 10% were designed. Sodium-silicate-based alkali activator provides an alkaline environment and acts as a key component to trigger the pozzolanic activity of the cementitious system. Test results indicate that both initial and final setting times decrease continuously with increasing alkali-activator dosage, while a slight rise in setting time occurs when the dosage exceeds 6% (Table 5 and Figure 4). Alkaline conditions accelerate OPC clinker hydration and prompt the rapid release of Ca^2+^ and OH^−^. Meanwhile, CaSO_4_ in PG dissociates, generating substantial AFt and C-S-H and greatly shortening the setting period. Nevertheless, excessive alkali-activator dosage leads to high-concentration sodium ions accumulating within the system. An adsorbed barrier layer forms on mineral particle surfaces, inhibiting ongoing hydration and resulting in a slight increase in setting time.

The mechanical test results reveal that the compressive and flexural strengths of the cementitious system keep increasing with rising alkali-activator dosage, and the growth tends to level off beyond 6%. Under alkaline conditions, Si-Al bonds in FA break down. Dissolved active Si and Al ions combine with calcium ions released from PG and OPC to form a continuous gel network, densify the microstructure and greatly improve mechanical strength. Once the alkali-activator dosage exceeds 6%, reactive minerals available for reaction are largely consumed. Excess alkali cannot further increase the total amount of hydration products, so strength growth slows down. Meanwhile, surplus alkali salts tend to crystallize inside the matrix, bringing potential risks to long-term volume stability [27]. Considering the peak mechanical performance, together with setting-related constructability, the optimal external alkali-activator dosage is determined as 6%.

#### 3.1.4. Modification Effect of RPP on Matrix Performance

**Figure 5:**
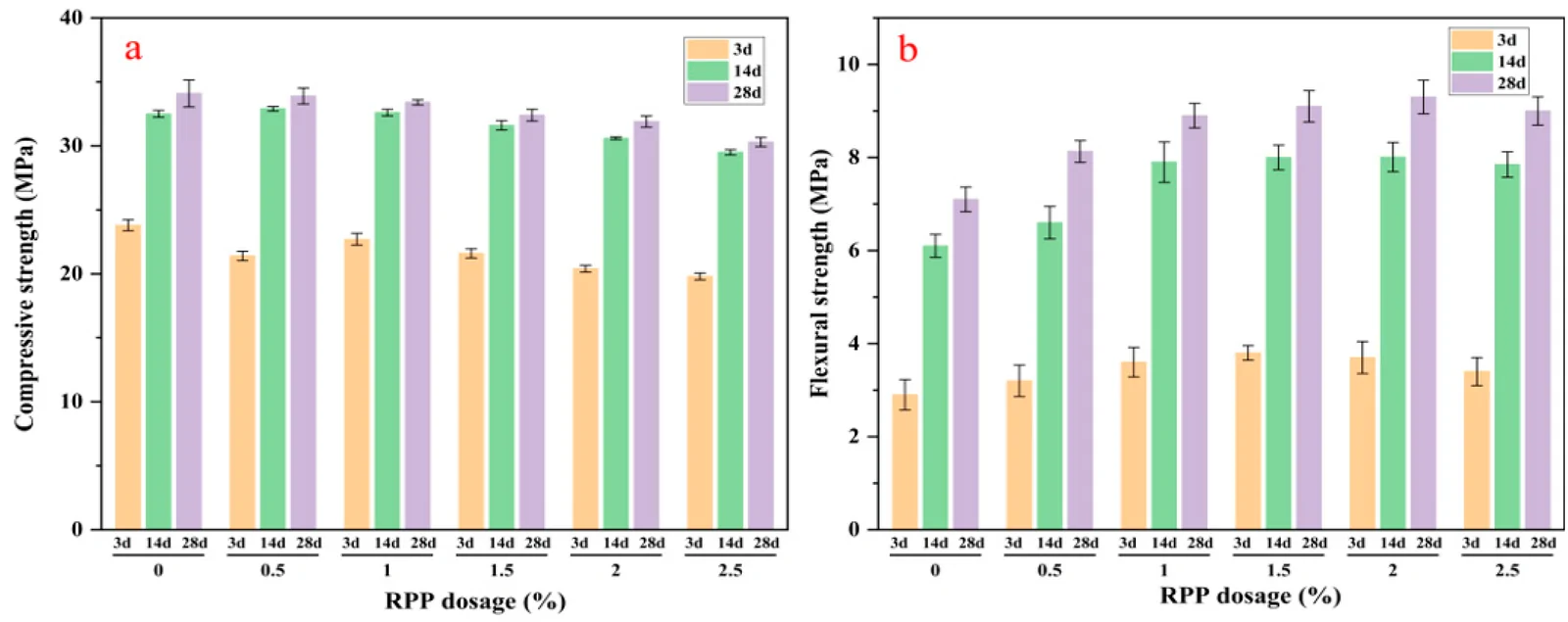
Effects of RPP dosage on mechanical strengths of cementitious materials: (**a**) compressive strength; (**b**) flexural strength.

As a polymer-based modifying admixture, RPP mainly improves the toughness and crack resistance of PG-based materials. Six dosage levels of RPP, ranging from 0% to 2.5%, were tested. The experimental data (Table 6 and Figure 5) reveal that both initial and final setting times increase synchronously with rising RPP content. Upon contact with water, polymer particles form a hydrophobic polymer film on mineral surfaces. This film blocks water–mineral contact, retards the hydration process and extends constructability time.

The mechanical properties exhibit obvious divergent trends: with the increase in RPP dosage, the compressive strength gradually decreases, while the flexural strength continuously rises. When the RPP dosage is lower than 1 wt.%, the 28 d compressive strength decreases from 36.7 MPa at 0 wt.% RPP to 36.4 MPa at 1 wt.% RPP; within the dosage range of 1–2.5 wt.%, the compressive strength further drops from 36.4 MPa to 30.7 MPa. The polymer film formed by hydrated RPP partially replaces the rigid inorganic hydration skeleton and reduces the compressive capacity of the matrix. The compressive strength shows only a slight reduction at low RPP dosage, while a more significant decline occurs when RPP dosage exceeds 1 wt.% because the inorganic cementitious skeleton is largely diluted. Nevertheless, the continuous flexible polymer network can disperse stress and hinder the propagation of microcracks, remarkably improving the flexural toughness, water resistance and crack resistance of the material. Test results show that the 28 d flexural strength is 7.1 MPa without RPP, and it increases to 8.9 MPa at an RPP dosage of 1 wt.%. The test results demonstrate that at an RPP dosage of 1%, the attenuation of compressive strength is limited, whereas the flexural performance is greatly enhanced. Balancing structural strength and toughness requirements, the optimal RPP dosage is determined as 1%. Under the conditions of water–cement ratio 0.4, alkali-activator dosage 6 wt.% and RPP dosage 1 wt.%, the 28 d compressive strength and flexural strength of the material are 36.4 MPa and 8.9 MPa, respectively.

#### 3.1.5. Micro-Hydration Mechanism Analysis of Optimally Proportioned Cementitious Matrix

**Figure 6:**
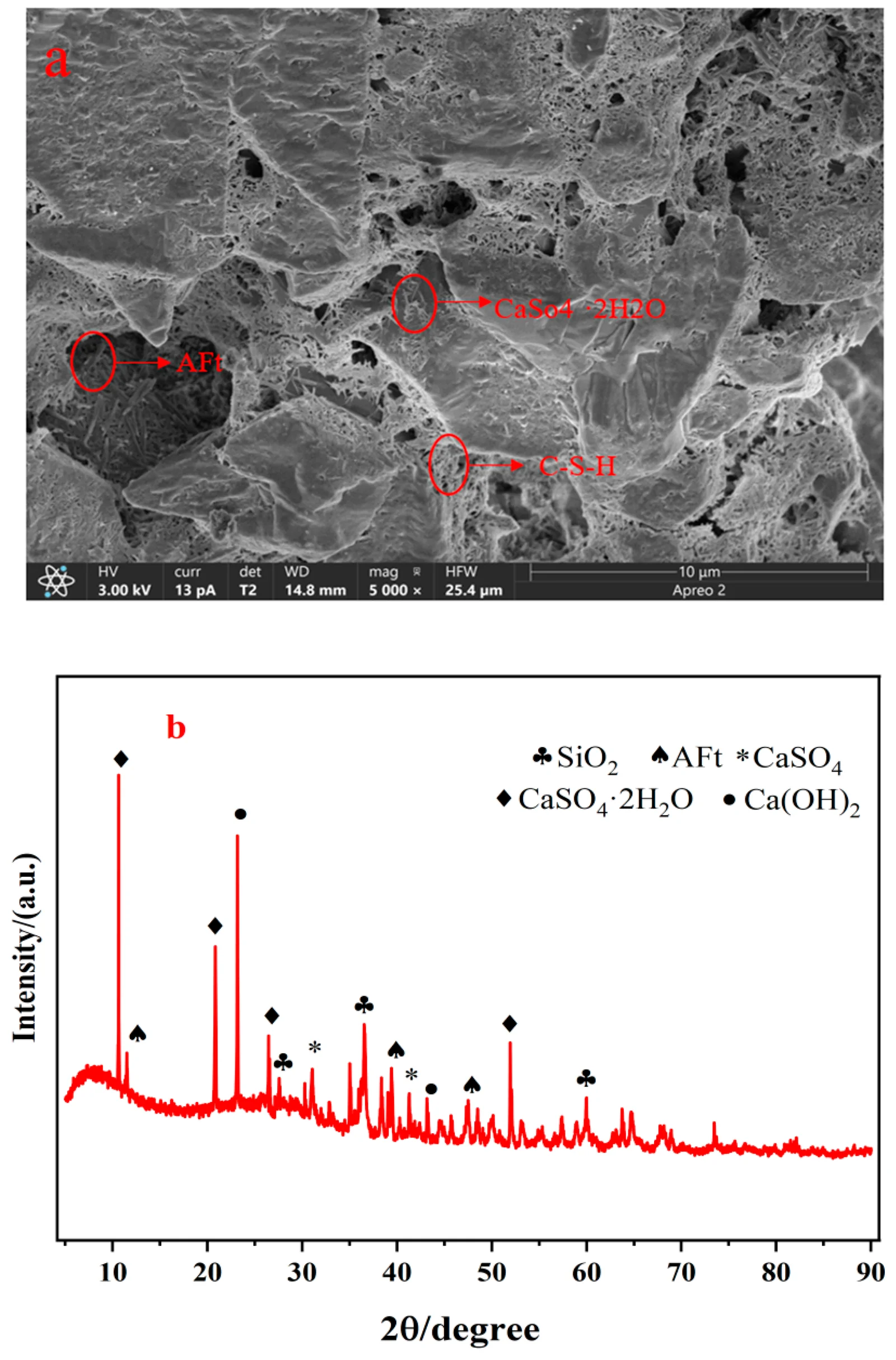
SEM and XRD analyses of PG-based cementitious materials: (**a**) SEM; (**b**) XRD.

SEM and XRD phase characterizations were performed on the optimally proportioned specimens cured for 28 d, and the results are presented in Figure 6. A slight porous feature can be observed in Figure 6a for the foaming-agent-free PG-based cementitious matrix. These intrinsic micro-pores stem from water consumption during hydration, volume variation caused by hydration-product precipitation and gas escape during paste hardening, rather than artificial foaming. Only 28-day-cured samples were characterized by SEM in this study. From the hydration mechanism perspective, early-age specimens (3 d) contain more unreacted phases and insufficient filling of hydration products, leading to relatively more micro-pores and lower mechanical strength. As curing age increases to 14 d and 28 d, ongoing pozzolanic reactions produce secondary AFt and C-S-H gels that fill micro-voids, refine pore structures and densify the matrix, contributing to continuous strength growth. The micro-morphology reveals an interlaced distribution of flaky calcium sulfate dihydrate and needle-like AFt. Flocculent C-S-H gel fills the interstices among crystals, and polymer films cover the surface of hydration products to construct an interwoven organic–inorganic composite skeleton.

XRD patterns verify that the main hydration products of the system are calcium sulfate dihydrate, AFt and C-S-H. Under alkali activation, the silicate-aluminate components of FA fully react, generating abundant AFt and C-S-H, which fill pores and improve matrix compactness. The continuous flexible films originated from RPP mitigate matrix brittleness and address the cracking defect commonly existing in high-content PG-based materials.

The complete hydration pathway is described as follows: OPC hydration produces calcium hydroxide. Combined with alkali activation, the pozzolanic reaction of FA is triggered. Calcium ions, sulfate ions and aluminum ions in the system react to form AFt and C-S-H. Meanwhile, polymers form films for a toughening effect, jointly constructing a composite cementitious matrix with high strength and high toughness.

(10) $$Ca{\left(OH\right)}_{2}+SiO_{2}\to \text{C-S-H}$$

(11) $$Ca{\left(OH\right)}_{2}+Al_{2}O_{3}+CaSO_{4}+H_{2}O\to AFt$$

### 3.2. Regulation of Porous PG-Based Filter Material Structure and Performance by Foaming Process

Based on the optimal mixing ratio of the cementitious matrix obtained above, three groups of experiments, including foaming-agent screening, foam content and mixing time, were carried out to regulate the pore structure, mechanical-abrasion resistance and water-absorption performance of the filter material.

#### 3.2.1. Performance Comparison and Screening of Four Types of Foaming Agents

**Figure 7:**
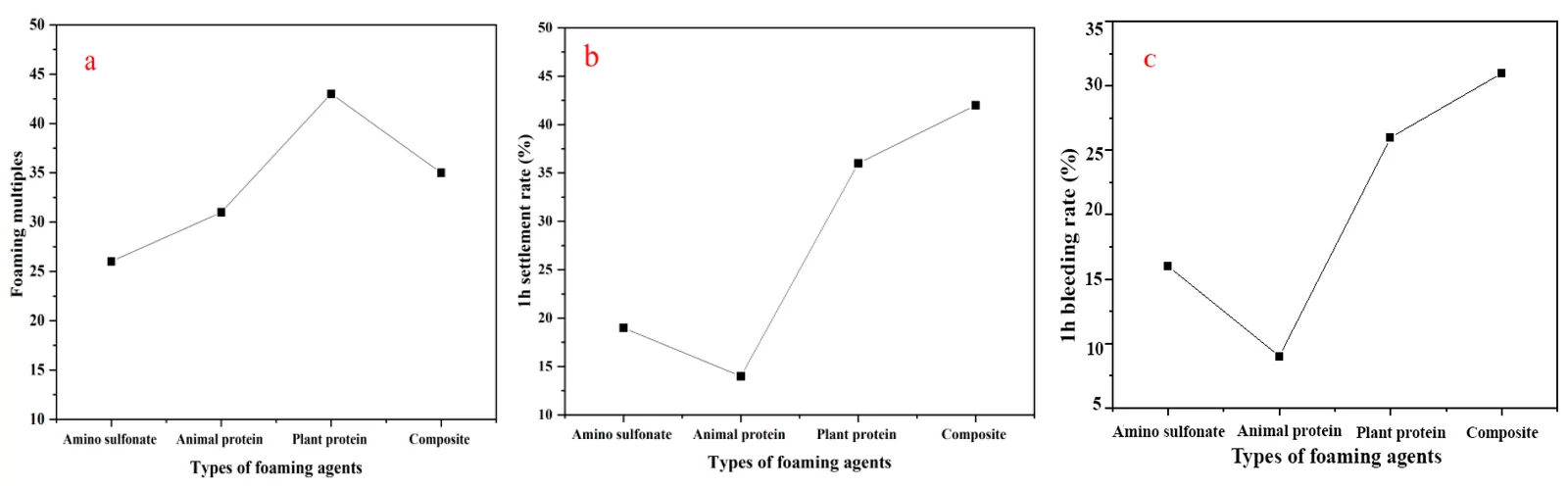
Performance comparison of different types of foaming agents: (**a**) foaming multiple; (**b**) 1 h settlement rate; (**c**) 1 h bleeding rate.

Four foaming agents, namely aminosulfonate, animal-protein, plant-protein, and composite type (prepared by mixing the above three agents at a mass ratio of 1:1:1), were compared. Foam stability was evaluated by three indices: foaming multiple, 1 h settlement rate and 1 h bleeding rate. The test results are shown in Figure 7.

The foaming multiple decreases in the following sequence: plant-protein > composite type > animal-protein > aminosulfonate. Plant-protein foaming agent exhibits the highest foaming capacity yet poor foam-film stability. It presents a 1 h settlement rate of 36% and a 1 h bleeding rate of 26%. A large number of foams rupture during static placement, leading to limited pores and non-uniform pore size after molding. The composite foaming agent shows the most severe foam collapse, with a 1 h settlement rate of 42% and a 1 h bleeding rate of 31%. The aminosulfonate agent only achieves a foaming multiple of 26, which causes high reagent consumption to satisfy identical pore-structure requirements.

By contrast, the animal-protein foaming agent delivers a moderate foaming multiple of 31, together with a 1 h settlement rate of 14% and a 1 h bleeding rate of 9%. Its dense liquid film resists liquid drainage, contributing to high foam retention during stirring and molding. Uniform interconnected mesopores can be obtained. Accordingly, animal-protein foaming agent is selected as the pore-forming raw material.

#### 3.2.2. Effects of Foam Content on Physical Properties of Filter Material

**Figure 8:**
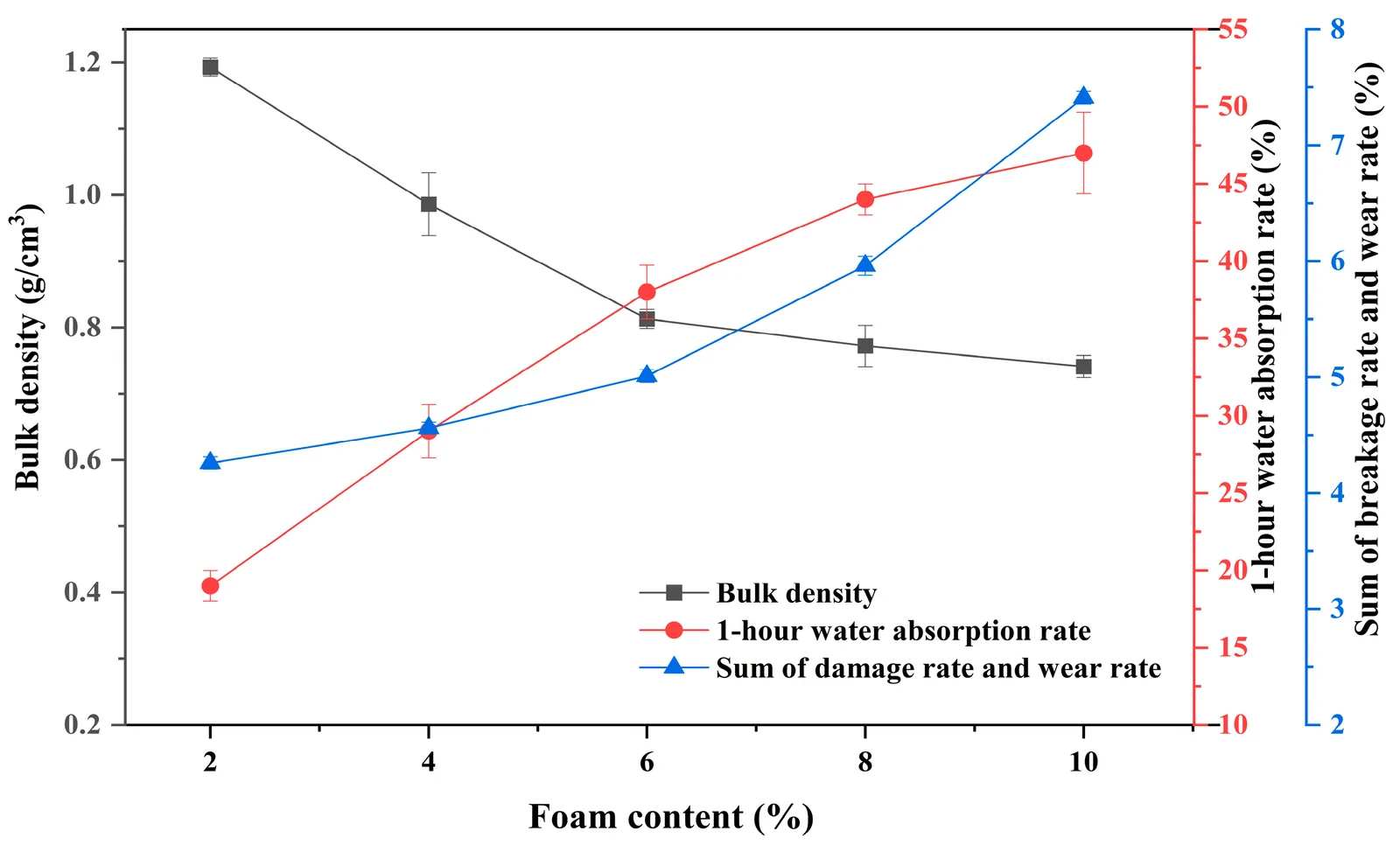
Influence of foam content on the physical properties of filter material.

The selected animal-protein foaming agent was adopted as the pore-forming agent. The mixing time and stirring rotational speed were fixed at 2 min and 400 rpm, respectively. External foam contents of 2%, 4%, 6%, 8% and 10% were designed according to mass ratio. The test results are presented in Figure 8.

With the increase in foam content, the bulk density of the filter material continuously decreases, while the 1 h water-absorption rate and breakage-abrasion rate rise simultaneously. In the foam content range of 2–6%, the bulk density declines markedly, pores develop rapidly and water-absorption channels are continuously enriched; the 1 h water-absorption rate increases from 20% to 38%. When the foam content exceeds 6%, excessive foam introduces abundant free water, the pore-wall thickness keeps decreasing, and the filter material presents a loose structure. The breakage-abrasion rate rises sharply from 5.01% to 7.41%, and the material tends to fragment under water-flow scouring, failing to meet the application requirements for water-treatment filter media.

At the foam content of 6%, the filter material achieves a bulk density of 0.813 g/cm^3^, a 1 h water-absorption rate of 38% and a breakage-abrasion rate of 5.01%. Balancing the lightweight porous characteristic and abrasion resistance, the optimal foam content is determined as 6%.

#### 3.2.3. Effects of Mixing Time on Pore and Mechanical Properties of Filter Material

**Figure 9:**
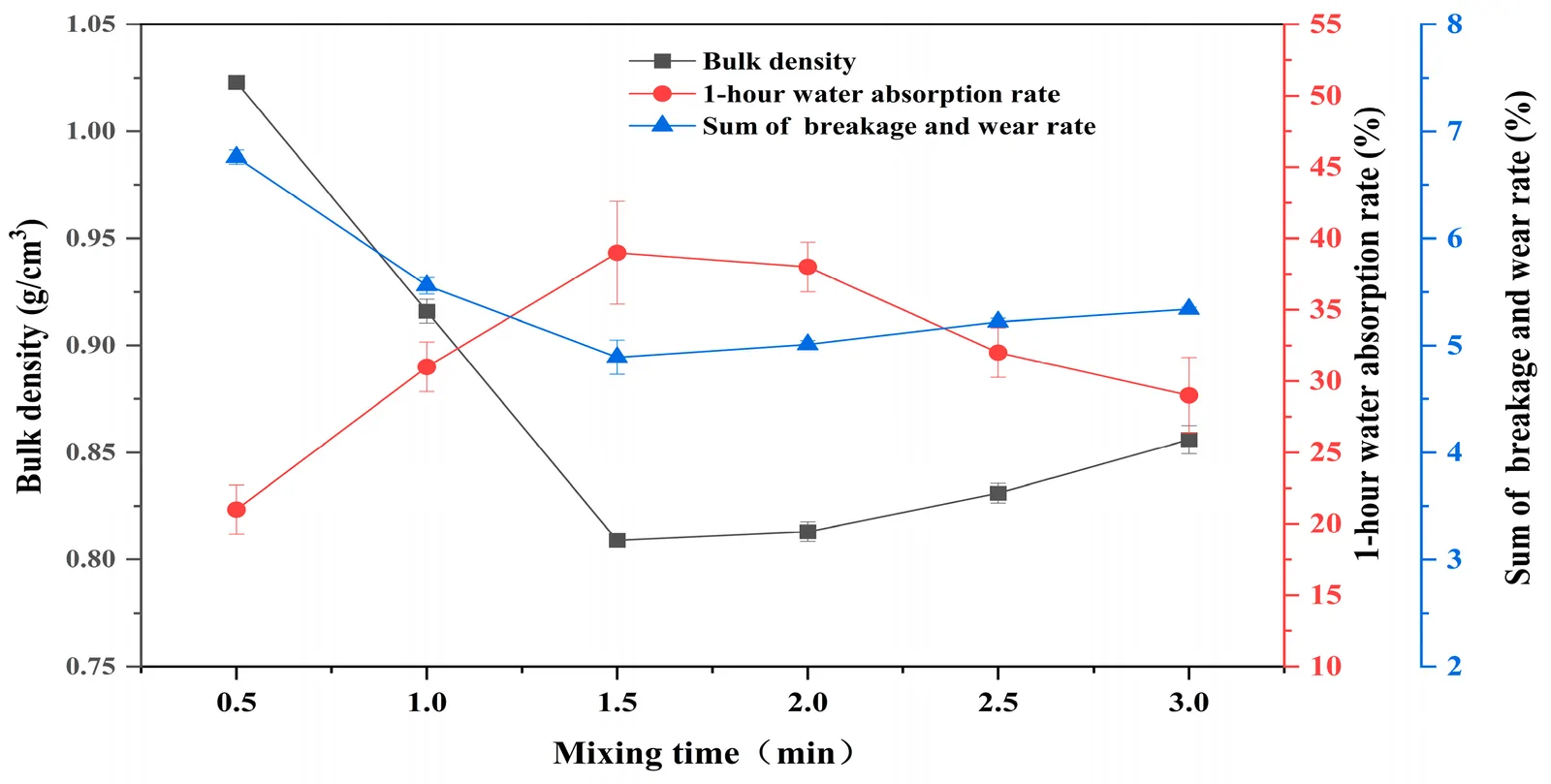
Influence of mixing time on pore and mechanical properties of filter material.

The foam content was fixed at 6% and stirring speed at 400 rpm, with stirring-time gradients ranging from 0.5 min to 3 min. The experimental results are presented in Figure 9. For stirring durations below 1.5 min, insufficient mixing occurred between paste and foam. Foam agglomeration created locally large pores in some regions, while other areas remained dense. This resulted in higher bulk density and breakage-abrasion rate, fewer pore channels and lower water-absorption rate. At 1.5 min mixing time, foams were well dispersed, and mesopores were homogeneously distributed. The bulk density reached its minimum of 0.809 g/cm^3^, the 1 h water-absorption rate peaked at 39%, and the breakage-abrasion rate was as low as 4.89%. When mixing time exceeded 1.5 min, sustained mechanical shearing damaged the liquid film of animal-protein foams. Many air bubbles ruptured, pore number decreased and water-absorption performance dropped. Pore walls became denser again, and both bulk density and breakage-abrasion rate increased. Therefore, the optimal mixing time was determined to be 1.5 min.

#### 3.2.4. Basic Performance and Leaching-Safety Evaluation of Optimal Filter Material

Filter media were prepared with the optimal binder formula and foaming process. The bulk density, 1 h water absorption and sum of breakage and wear rates were measured as 0.809 g/cm^3^, 39% and 4.89%, respectively, which meet the requirements specified in CJ/T 299-2008. BET measurements reveal that the filter material possesses a specific surface area of 20.151 m^2^/g and an average pore size of 6.93 nm. Dominated by mesoporous structure, it can provide sufficient sites for phosphate adsorption.

Leaching-toxicity tests were carried out according to GB 5085.3-2007. Measured concentrations of copper, zinc, cadmium, lead, chromium, arsenic and fluoride were all well below the standard limits (Table 7). Leaching rates were calculated from mass-weighted elemental fractions of the three raw materials (50 wt.% PG, 15 wt.% FA, 35 wt.% OPC), assuming no elemental loss during mixing and curing. The mass-based leaching rates of Pb, Cr, Ba and P were all below 0.1%, while fluoride reached a relatively high leaching rate of 1.284%.

These results agree with reported data for phosphogypsum-based cementitious materials [6,7]. In slag- and aluminate-dominated alkali-activated solid-waste binders, heavy-metal cations are effectively immobilized via hydroxide precipitation, ion exchange and physical encapsulation by C-(A)-S-H gel and ettringite, yet anionic fluoride cannot be completely stabilized. For the optimal PG-slag-calcium-carbide-residue formulation, good heavy-metal retention was obtained under simulated acid-rain conditions, but fluoride leaching still reached 2.173 mg/L [7].

Published work shows organic polymers act as auxiliary modifiers to improve heavy-metal immobilization of cementitious systems. At optimal dosages, super-absorbent polymer (SAP) reduces lead leaching by 47–75% through ion-trapping within hydrogel networks [28]. Polycarboxylate superplasticizer indirectly lowers heavy-metal and phosphorus dissolution by refining matrix pore-size distribution. RPP was incorporated in this study. It develops continuous thin films at the late hydration stage, mainly for improving material toughness, flexural strength and water resistance, while partially blocking micro-scale matrix percolation pathways to mitigate pollutant leaching. Nevertheless, polymers cannot fundamentally eliminate fluoride release from soluble salts present in raw PG. Raw PG contains soluble fluoride-bearing salts and phosphorus-rich impurities. After hydration and solidification in the PG–FA–cement system, fluorite (CaF_2_) exhibits inherent solubility, and hydration products have limited lattice-substitution capacity for fluoride ions [1]. Interconnected pores in the porous material increase contact between leachant and soluble impurity phases, so fluoride dissolution cannot be fully suppressed [6,29]. Though the filter media satisfies GB 5085.3-2007 leaching-toxicity requirements, its fluoride leaching concentration (1.027 mg/L) is close to Class III surface-water (GB 3838-2002) and Class III groundwater (GB/T 14848-2017) limits, and phosphorus also poses a potential over-limit risk under these conditions.

### 3.3. Static Phosphorus-Adsorption Performance of PG-Based Filter Material

#### 3.3.1. Phosphorus Removal Performance Comparison of Different Filter Materials

**Figure 10:**
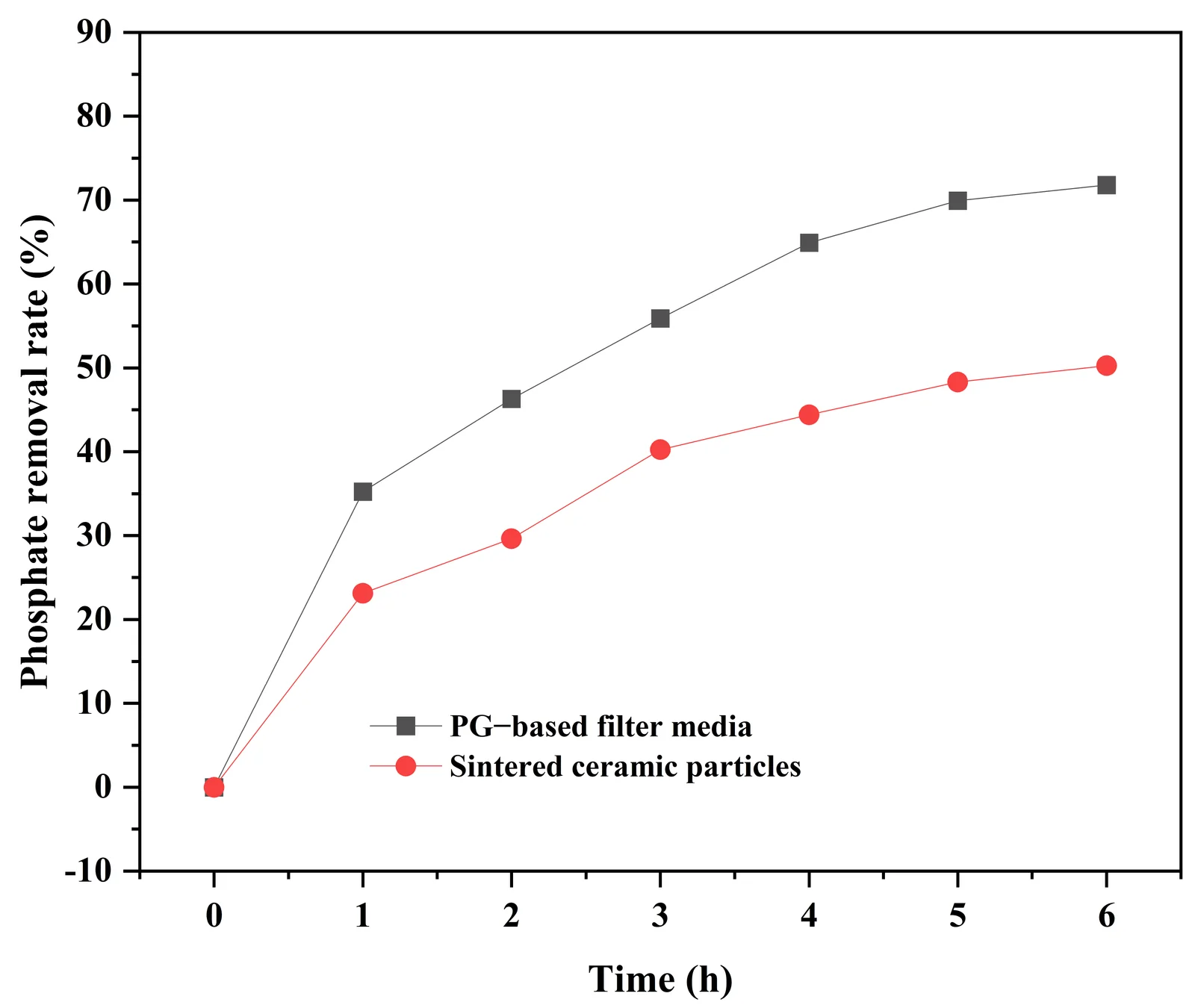
Comparison of phosphorus-removal performance between PG-based filter media and sintered ceramsite.

Simulated wastewater with an initial total phosphorus concentration of 40 mg/L was prepared, and a 6 h static adsorption test was conducted using the self-prepared PG-based filter material and commercial sintered ceramsite, respectively. The test results are displayed in Figure 10. The phosphorus removal rates of the two filter materials increased continuously with shaking time throughout the test. The adsorption rate was the highest within the initial 0–1 h, slowed down after 4 h, and basically reached adsorption equilibrium at 6 h. At the end of the test, the phosphate-removal efficiency of the sintered ceramsite was only 50.28%, while the phosphate-removal efficiency of the PG-based filter media reached 71.81% at 6 h. Statistical analysis was performed to quantify the difference in phosphate-removal performance (Table 8). Levene’s test verified the homogeneity of variances, and independent-samples t-test results indicated that the PG-based filter media exhibited significantly superior phosphate-removal capacity compared with sintered ceramic particles (mean difference = 0.7207, *p* < 0.00001, 95% CI: 0.3955–1.0458). Considering both phosphate-removal performance and preparation cost, the cold-bonded PG-based filter material prepared in this study may provide a feasible option for practical engineering applications.

#### 3.3.2. Phosphate-Removal Kinetic Analysis

**Figure 11:**
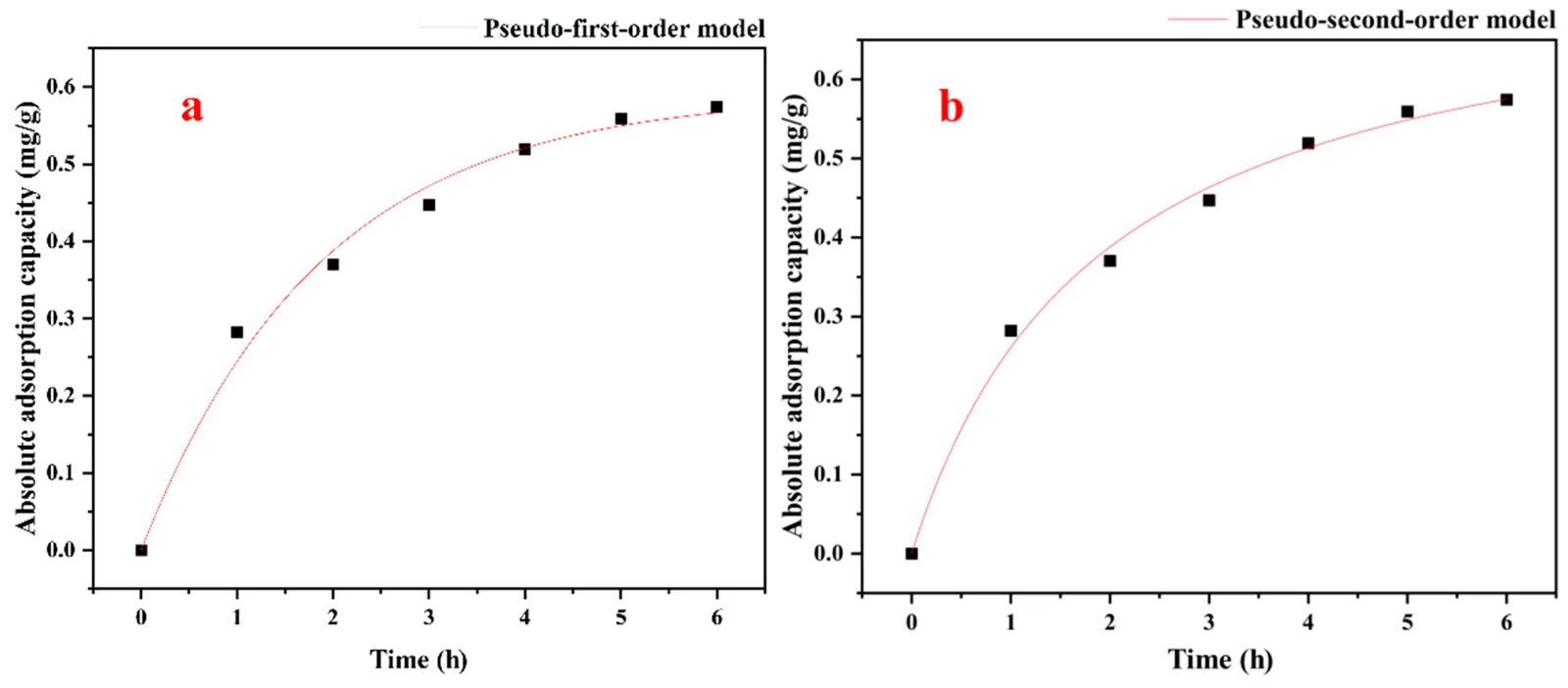
Kinetic fitting results for phosphate adsorption onto PG-based filter media: (**a**) Pseudo-first-order model; (**b**) pseudo-second-order model.

Static adsorption experimental data were fitted by pseudo-first-order and pseudo-second-order kinetic models to analyze the phosphate adsorption rate of PG-based filter media. The fitting parameters and curves are listed in Table 9 and Figure 11.

It should be emphasized that phosphate elimination by PG-based filter media arises from multiple pathways, including physical surface adsorption, calcium-phosphate chemical precipitation and immobilization. The capacity derived from static batch tests corresponds to the apparent equilibrium capacity, integrating contributions from both surface adsorption and insoluble phosphate precipitation. Both models exhibited high correlation coefficients for the adsorption process. The pseudo-first-order model produced a calculated apparent equilibrium capacity of 0.59 mg/g, which was close to the experimental value of 0.57 mg/g, with an *R*^2^ of 0.987. The pseudo-second-order model had a higher *R*^2^ of 0.995, while its calculated apparent equilibrium capacity of 0.76 mg/g deviated from the measured value. The fitting results indicate that the overall phosphate-removal process is mainly governed by chemical interactions (calcium-phosphate precipitation-dominated), and physical diffusion also plays an important role throughout the removal process.

### 3.4. Micro-Characterization Analysis of Filter-Media Structure and Phosphate-Removal Mechanism

#### 3.4.1. Pore-Structure Analysis via N_2_ Adsorption–Desorption

**Figure 12:**
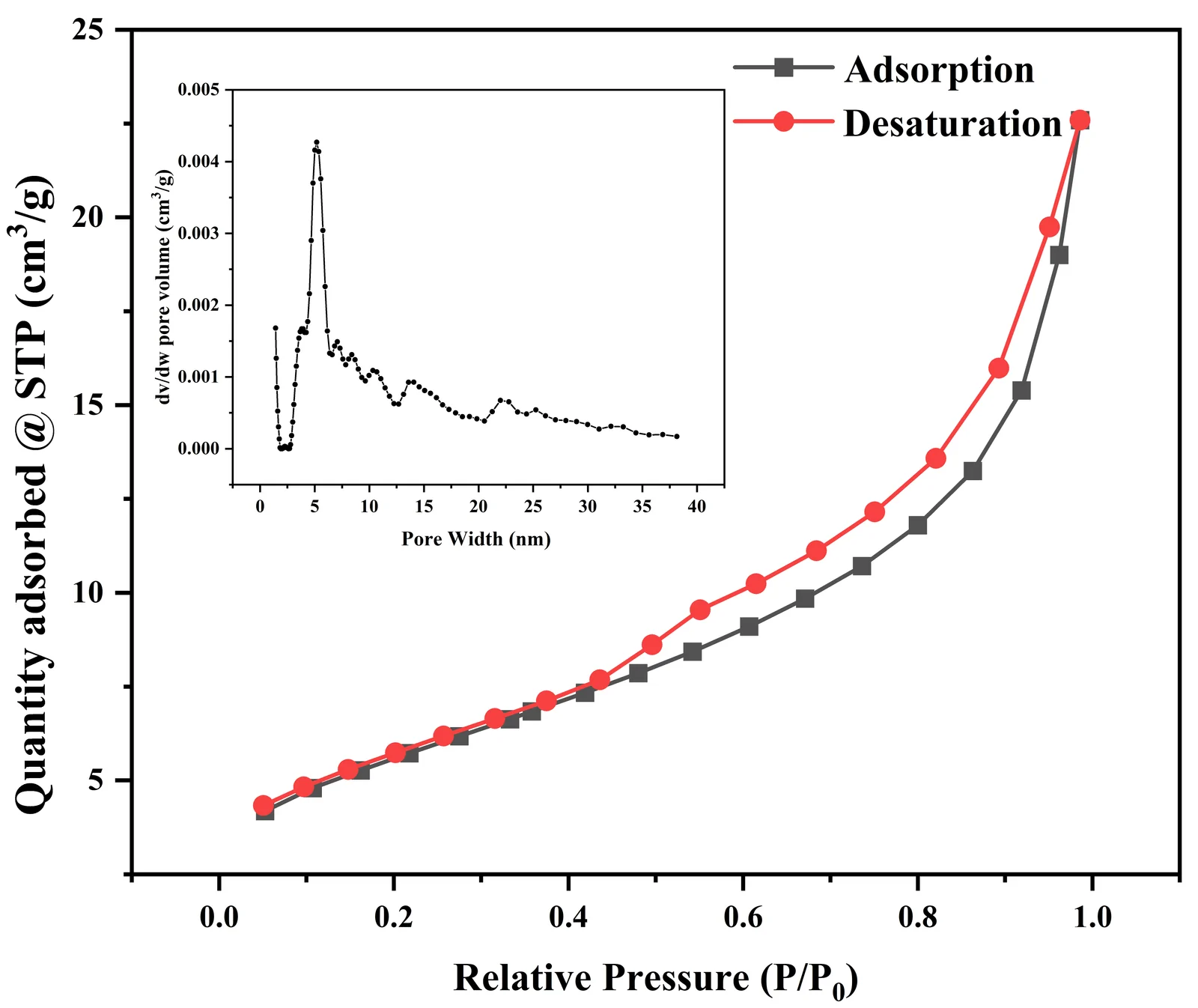
N_2_ adsorption–desorption isotherms and pore-size distribution.

N_2_ adsorption–desorption tests are used to quantitatively analyze the specific surface area, pore volume, and pore-size distribution of filter media, serving as the structural basis for interpreting the adsorption capacity of the material. Before low-temperature nitrogen adsorption–desorption tests, the porous filter media were crushed and sieved to collect particles of 0.25–0.5 mm. The sieved samples were vacuum-degassed at 105 °C for 12 h to remove adsorbed water and volatile impurities. The BET model was applied to calculate the specific surface area within the appropriate pressure range of the adsorption isotherm, while the BJH method was adopted to determine pore volume and pore size distribution. The tested sample exhibited a specific surface area of 20.151 m^2^/g, total pore volume of 0.035 cm^3^/g and average pore size of 6.93 nm. The adsorption–desorption isotherm in Figure 12 belongs to the Type IV isotherm, with an obvious hysteresis loop at medium-high relative pressure [30], revealing that mesopores dominate its inner structure.

Monolayer and multilayer nitrogen adsorption takes place in the low-pressure region (*P*/*P*_0_ = 0–0.4), corresponding to the filling of micropores and small-sized mesopores. The hysteresis loop within a *P*/*P*_0_ of 0.4–1.0 originates from capillary condensation inside mesopores, demonstrating abundant interconnected open pores. Phosphate ions in water can readily diffuse into interior pores rather than merely attaching to the outer surface.

A characteristic peak occurs in the pore-size distribution curve between 5 and 8 nm. Mesopores in this size range prevent ion-diffusion retardation that arises from too-small pores, and they also avoid the loss of specific surface area and adsorption sites brought about by excessively large pores. The continuous mesoporous network forms the structural foundation for the good phosphate-removal performance of the filter media.

#### 3.4.2. Comparison of SEM Micro-Morphologies Before and After Phosphorus Adsorption

**Figure 13:**
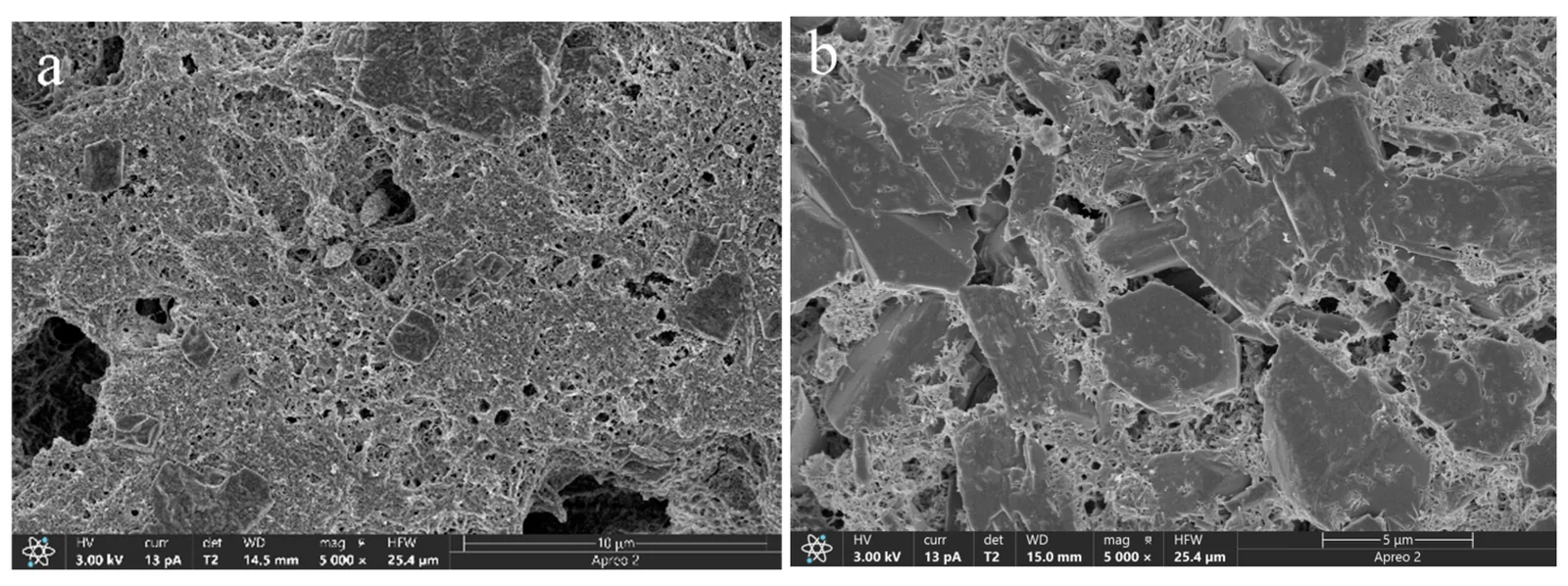
SEM images of PG-based filter media before and after phosphate adsorption: (**a**) Before adsorption; (**b**) after adsorption.

SEM observations at 5000-fold magnification were performed on filter media before and after adsorption to intuitively reveal the phosphate adsorption and precipitation mechanisms, as illustrated in Figure 13. Before adsorption, the sample surface exhibited an undulating texture with interlaced block-like calcium sulfate dihydrate crystals.

Numerous interconnected irregular pores with a wide size range were generated among crystal gaps and inside the matrix, constructing multi-level three-dimensional mass-transfer channels. The rough crystal surface was covered with flocculent C-S-H gels and polymer films, and abundant micro-protuberances further raised the local specific surface area to supply sufficient active adsorption sites.

After 6 h of phosphorus-removal adsorption, obvious changes occurred in micro-morphology. A large number of originally open, interconnected pores were filled and blocked by newly formed solid products, and the pore size decreased evidently. Dense granular and acicular composite precipitates uniformly coated the crystal surface, and these precipitates aggregated into sheet-like layers in partial regions. Such precipitates corresponded to calcium phosphate crystals yielded by the reaction between calcium ions and phosphate radicals, confirming the proceeding chemical-precipitation process. This also accounts for the slight drop in water absorption and porosity after adsorption equilibrium.

Moreover, the crystalline skeleton of the matrix remained intact without dissolution or collapse after adsorption. It demonstrates that the PG-based cementitious skeleton possesses favorable chemical stability. Adsorption reactions mainly take place inside pore channels rather than destroying the overall material structure, which supports its recyclable application.

#### 3.4.3. Quantitative Elemental Analysis by XRF

XRF quantitative elemental measurement was carried out to identify the basic chemical components of filter media and provide material-level evidence for the continuous calcium-ion-release mechanism. The test results are summarized in Table 10. The mass fractions of CaO and SO_3_ in the filter media were 40.84% and 37.56%, respectively. These two components accounted for nearly 80% of the total composition, which indicates a typical calcium-sulfate-dihydrate cementitious system. The subsequent SiO_2_ (11.92%) and Al_2_O_3_ (5.63%) originated from fly ash and cement. Under alkali activation, they formed stable Si-Al-O frameworks that guarantee the mechanical strength and chemical stability of filter media. Trace impurities, including Fe_2_O_3_, MgO and TiO_2_, were inherited from raw materials, and their low contents would not bring adverse effects.

During immersion in an aqueous environment, calcium sulfate dihydrate reaches a reversible dissolution-equilibrium state and continuously releases free Ca^2+^ into water, which supplies sufficient cations for calcium-phosphate precipitation. Silicon-aluminum oxides possess high chemical resistance against aqueous corrosion, maintaining long-term structural integrity of filter media and enabling stable long-term phosphate-removal performance.

#### 3.4.4. XRD Phase-Evolution Analysis

**Figure 14:**
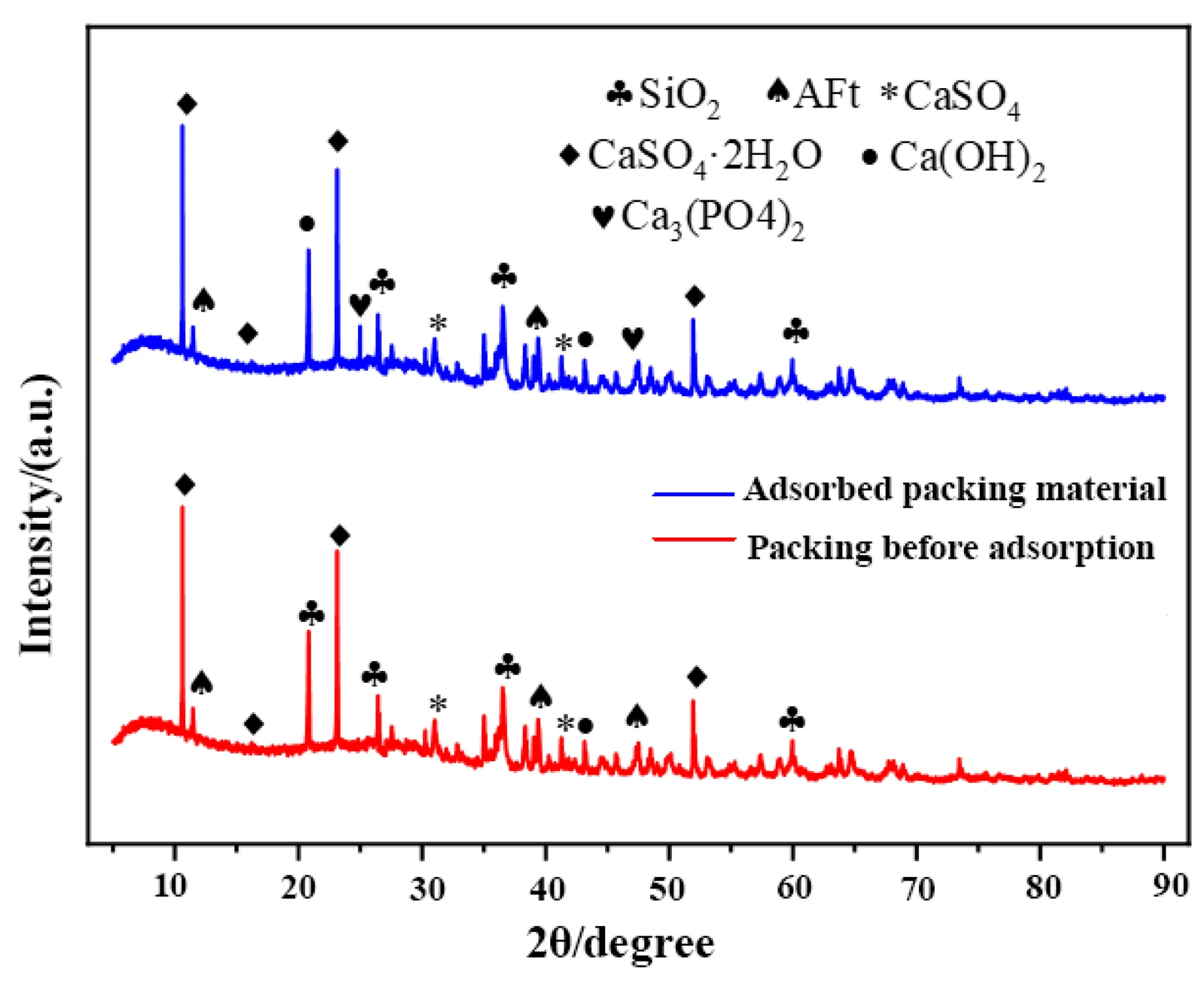
XRD patterns of PG-based filter media before and after phosphate adsorption.

XRD patterns were compared to identify crystal-phase variations before and after adsorption so as to illustrate the chemical-precipitation-dominated phosphorus-removal mechanism from the phase perspective (Figure 14). Before adsorption, the characteristic diffraction peaks of filter media corresponded to calcium sulfate dihydrate, AFt, quartz (SiO_2_), and trace calcium hydroxide, while no diffraction peaks assigned to phosphorus-containing crystals were detected. After 6 h static adsorption, distinct new diffraction peaks of Ca_3_(PO_4_)_2_ appeared at specific 2θ angles.

Meanwhile, the peak intensity of calcium sulfate dihydrate obviously decreased, accompanied by slight peak broadening. These observations demonstrate that calcium sulfate dihydrate continuously dissolves during immersion. The released Ca^2+^ combines with aqueous PO_4_^3−^ to form insoluble calcium-phosphate crystals, which stably deposit inside filter-media pores and serve as the main immobilized form of phosphorus. The diffraction peaks of AFt and quartz hardly change, indicating that silicate-aluminate hydration products and inert FA minerals do not participate in phosphorus-removal reactions. They merely act as a stable supporting skeleton without interfering with phosphate-precipitation processes. It further confirms that the core phosphorus-removal reaction originates from chemical precipitation between Ca^2+^ and phosphate radicals [31].

#### 3.4.5. FT-IR Functional Group Characterization Analysis

**Figure 15:**
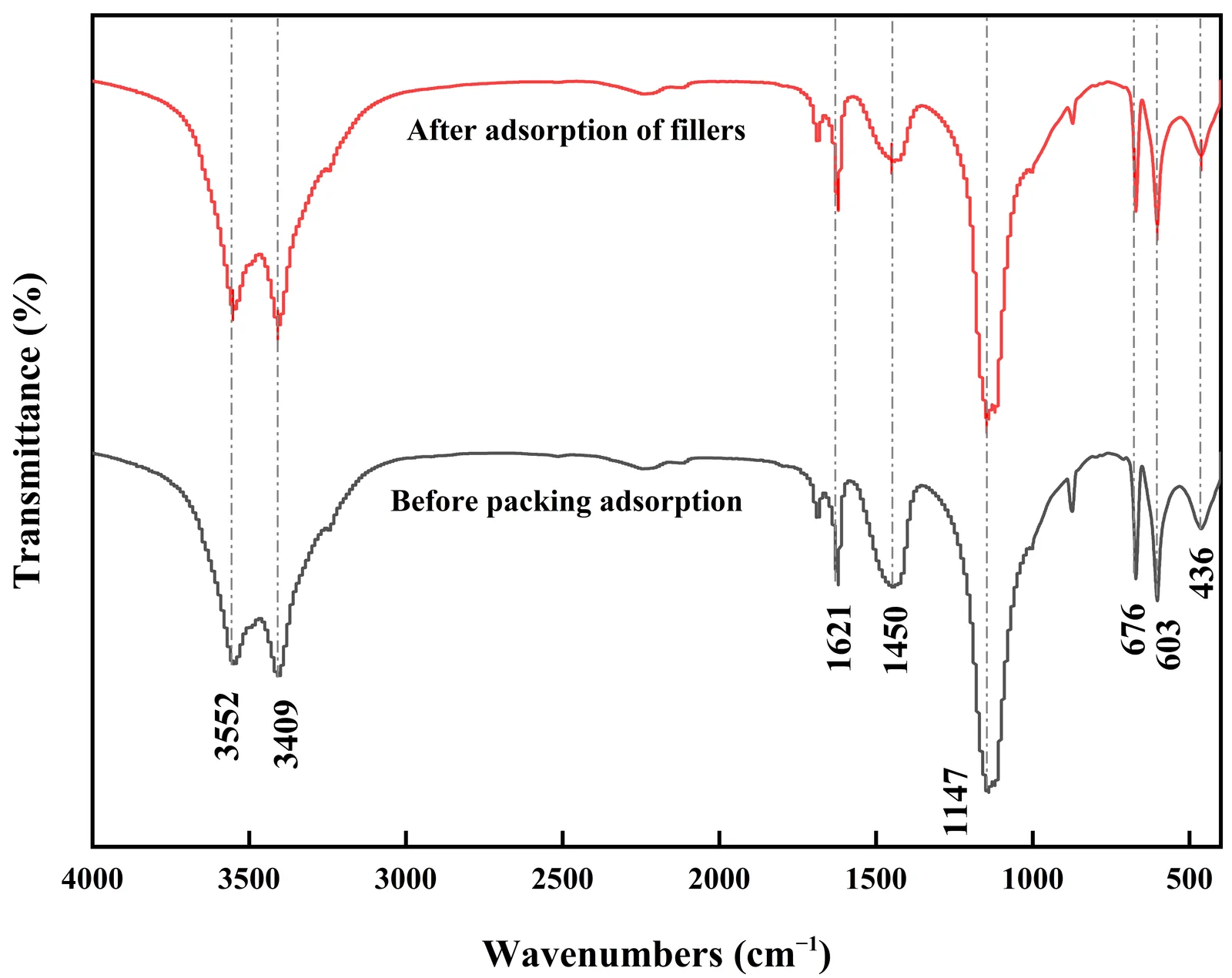
FT-IR spectra of PG-based filter media before and after phosphorus adsorption.

Fourier transform infrared spectroscopy (FT-IR) was used to analyze the variations in functional groups of the material before and after adsorption so as to evaluate the skeleton stability and ion-interaction modes of the matrix (Figure 15). The identified characteristic peaks are located at 3552, 3409, 1621, 1450, 1147, 676, 603 and 436 cm^−1^, as labeled in the figure. The broad absorption peaks at 3552 cm^−1^ and 3409 cm^−1^ are attributed to the stretching vibration of crystal water and hydroxyl (–OH) groups. No obvious offset in peak position and intensity was observed before and after adsorption, indicating that the crystal water structure inside the material was stable and hydroxyl groups did not participate in phosphate complexation reactions. The absorption peaks at 1621 cm^−1^ and 1450 cm^−1^ correspond to the stretching vibration of C=O double bonds. Slight peak shifts occurred after adsorption, demonstrating that a small amount of aqueous phosphate radicals underwent weak complexation adsorption with oxygen-containing functional groups on the surface of polymer films, which served as an auxiliary adsorption pathway.

The strong absorption peak at 1147 cm^−1^ is the characteristic vibration peak of Si-O-Si and Si-O-Al covalent bonds. The peak shape and position remained completely unchanged before and after adsorption, proving that the silicon-aluminum inorganic skeleton formed by FA and OPC exhibited no hydrolysis or corrosion in phosphorus-rich aqueous environments and possessed a stable chemical structure. The characteristic bending vibration peaks of Si-O at 676 cm^−1^, 603 cm^−1^, and 436 cm^−1^ showed no significant changes throughout the adsorption process.

Based on the FT-IR results, the polymer and silicon-aluminum matrix only serve as adsorption carriers and mass-transfer channels and do not participate in the main reaction. Phosphate removal from water is dominated mainly by chemical precipitation of calcium ions, whereas weak surface functional-group complexation and mesoporous physical adsorption only play auxiliary enhancement roles [32,33].

#### 3.4.6. Comprehensive Interpretation of Phosphate-Removal Mechanism for PG-Based Filter Media

**Figure 16:**
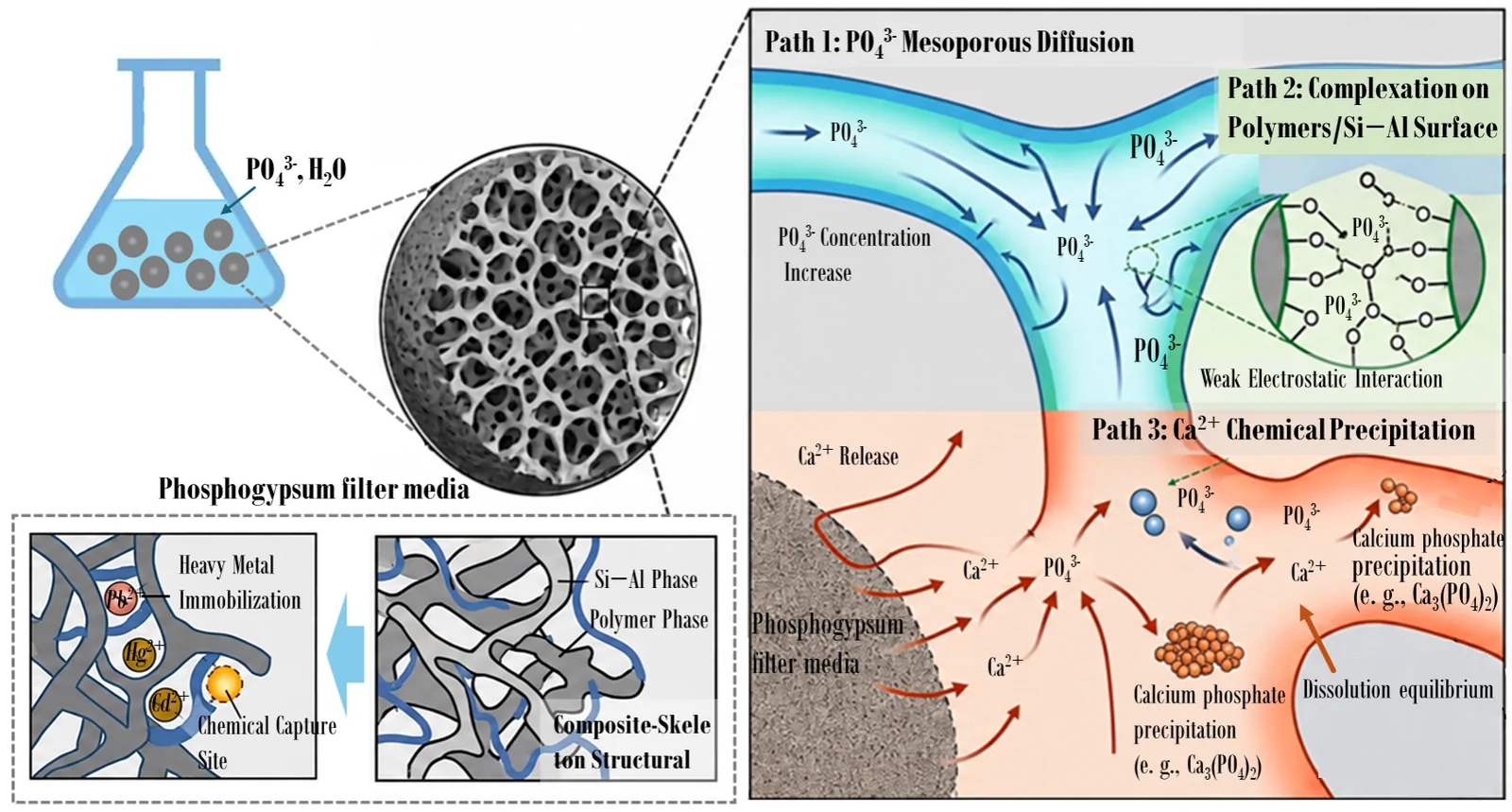
Phosphate-removal mechanism of PG-based filter media.

Based on multi-scale characterization (BET, SEM, XRD, XRF, FT-IR) and static-adsorption test data, the phosphate-removal performance of PG-based filter media relies on the synergistic effects of mesoporous physical adsorption, surface functional group complexation, and calcium-dominated chemical precipitation, among which chemical precipitation serves as the dominant pathway (Figure 16) [6,7]. The whole removal process can be divided into three stages.

Mesoporous physical adsorption: Driven by concentration gradients, phosphate ions diffuse inward through the interconnected mesoporous channels of the filter media. The porous structure effectively prolongs ion residence time and accumulates PO_4_^3−^ within internal pores, increasing local phosphate concentration and providing favorable conditions for subsequent precipitation reactions [1].

Surface functional group complexation (auxiliary): Polymer films and silicon-aluminum oxides on the filter media surface contain hydroxyl functional groups. A small portion of phosphate ions is immobilized via intermolecular forces and weak ionic bonding, playing a secondary and auxiliary role in phosphorus removal.

Calcium-dominated chemical precipitation (dominant): Calcium sulfate dihydrate in the filter media continuously dissolves and releases Ca^2+^ into pore water. The released calcium ions react with the pre-enriched PO_4_^3−^ to form poorly soluble Ca_3_(PO_4_)_2_ crystals, which stably deposit and fix phosphorus inside the pore channels, realizing permanent phosphate immobilization [7].

Silicon-aluminum hydration products and polymer components construct a stable supporting skeleton, which guarantees the structural stability and abrasion resistance of the filter media without consuming calcium ions or interfering with the dominant precipitation reaction. Different from commercial sintered ceramsite that merely relies on single-surface physical adsorption, the prepared PG-based filter media achieves superior phosphorus-removal capacity through sustainable calcium release and in situ precipitation. The precipitated products exhibit good chemical stability under neutral-to-alkaline aqueous conditions, thereby enabling the feasible application of the filter media in high-phosphorus wastewater treatment, constructed wetland systems, and rainwater purification. However, potential phosphorus and fluoride release risks may occur under acidic environments or long-term complex water field conditions, restricting its direct universal engineering application.

When compared with representative phosphate-removal materials, obvious differences can be observed in raw-material composition, structural parameters and dominant mechanisms. The commercial sintered clay ceramsite used in this study is a clay-based granular filter with an apparent density of 1.2 g/cm^3^ and a specific surface area of 8.75 m^2^/g. It only reached a phosphate-removal efficiency of 50.28% under identical test conditions, with phosphate removal dominated by physical pore-surface adsorption. Our previously reported non-sintered foamed fly-ash-based filter media is prepared with a fly ash-cement system via animal-protein foaming without high-temperature calcination; it has a bulk density of 762.8 kg/m^3^ and specific surface area of 8.75 m^2^/g, and its phosphorus removal originates from combined ion-exchange and surface precipitation, reaching 66.58% phosphorus removal under a 40 mg/L initial-phosphorus-concentration condition [34]. Phosphogypsum-fly ash sintered ceramsite (PFC), reported by [16], is fabricated by high-temperature sintering at 900 °C. Its phosphate immobilization is dominated by irreversible calcium-phosphorus crystal precipitation (hydroxyapatite and brushite), with a maximum adsorption capacity of 21.97 mg P/g. Nevertheless, high-temperature sintering brings relatively large energy consumption. Crystalline La-Al co-loaded Fe_3_O_4_ magnetic adsorbent developed by Long et al. is a rare-earth-modified magnetic powder adsorbent. It possesses a high specific surface area of 83.71 m^2^/g and ultra-high saturated phosphate adsorption capacity of 94.3 mg P/g, with ligand exchange as the main mechanism [13]. However, it depends on rare-earth chemical reagents and is difficult to apply as bulk granular filter media in practical filtration systems.

Compared with the above-mentioned materials, the present PG-based filter media adopts a low-energy cold-bond-forming route using bulk PG solid waste as the main raw material. It combines physical pore-surface adsorption and calcium-phosphate precipitation-dominated removal pathways. Although its apparent phosphate capacity is not as high as that of the rare-earth-modified magnetic adsorbent, it avoids high-temperature sintering or expensive rare-earth reagent inputs, and it exhibits superior phosphate-removal performance (71.81% after 6 h test) compared with commercial sintered clay ceramsite. It provides a feasible low-cost option for phosphorus-laden wastewater remediation, accompanied by large-scale PG resource utilization. Although the phosphate-removal performance of the PG-based porous filter media prepared in this work is still moderate, further surface-modification strategies will be considered in our follow-up research to improve its phosphate-removal capacity. Meanwhile, we will expand performance verification and optimization targeting other water-quality indicators, such as COD and ammonia-nitrogen, for various real-wastewater scenarios.

## 4. Discussion

### 4.1. Regulation Mechanism of RPP-Synergized Alkali Activation on PG-Based Composite Matrix

In this study, RPP was incorporated into a high-content (50 wt.%) PG-based alkali-activated cementitious system, which is distinct from previous studies focusing on pure inorganic gypsum cementitious materials. The hydration of OPC-PG-FA systems is dominated by rigid crystal networks of C-S-H and AFt, which endow the matrix with high brittleness. Such materials are prone to penetrating cracks under compression, resulting in a high damage rate of filter media under long-term water scouring.

After incorporating 1 wt.% RPP, the polymer emulsion dehydrates and forms continuous films during hydration. The formed polymer films coat calcium sulfate dihydrate, FA vitreous particles, and AFt crystals, constructing an organic toughening network within the rigid inorganic skeleton.

In terms of setting performance, the polymer film temporarily hinders the contact between water and cementitious particles. Thus, both initial and final setting times are prolonged with the increase in RPP dosage, which benefits on-site mixing and casting construction. Nevertheless, when the RPP dosage exceeds 1 wt.%, the continuous organic phase dilutes the proportion of hydration gels, leading to a continuous decline in compressive strength while only slightly improving flexural toughness.

The activation effect of alkali activator synergizes well with polymer modification. Water glass provides abundant OH^−^ to break the silicon-oxygen and aluminum-oxygen network of FA, releasing active silicon and aluminum ions. These ions rapidly react with Ca^2+^ leached from PG to form high-strength AFt and low-calcium C-S-H gels. The rigid inorganic skeleton provides stable mechanical support for polymer films and avoids excessive strength degradation caused by excessive organic incorporation.

This work realizes stable molding at a high PG dosage of 50 wt.%. The dual modification strategies of secondary FA hydration and polymer toughening effectively solve the bottlenecks of insufficient strength and easy cracking of high-gypsum systems. This study provides a feasible regulation strategy for the large-scale resource utilization of PG as cementitious material. By coupling secondary FA hydration and RPP-induced toughening, this study achieved stable fabrication of cementitious matrices with a PG dosage of 50 wt.%. The dual modification strategy overcomes the low strength and high cracking susceptibility that commonly plague conventional high-dosage PG cementitious materials. This work offers a feasible regulation route to promote the bulk utilization and disposal of PG in cementitious systems.

### 4.2. Mechanism of Coupled Foaming-Polymer Effects on Pore Structure of Porous Filter Media

Foaming-agent screening tests showed that the animal-protein foaming agent showed the best overall stability. Sulfamate-based and composite foaming agents had serious foam bleeding and sedimentation. Large amounts of foam ruptured during molding, so interconnected mesoporous channels could hardly be formed. With long-chain molecular structures, protein molecules build dense protective films at the gas–liquid interface, lower the foam drainage rate, and adapt to the slow-hydration features of the PG-based matrix.

There is a critical equilibrium range for foam content and mixing time. When the foam content was below 6%, the system produced insufficient pores and low specific surface area, thus providing limited adsorption sites for phosphate. Once the foam content exceeded 6%, the internal pore walls became too thin, and the breakage-and-abrasion rate exceeded the 6% standard limit under water flow impact. Mixing time shorter than 1.5 min led to foam agglomeration and uneven pore-size distribution. If mixing time went above 1.5 min, mechanical shearing damaged protein-based foam films, and the number of mesopores obviously dropped.

RPP had dual functions in this system. On one hand, polymer films wrapped crystals on pore walls and improved the anti-scouring capacity of pore walls. At the same foam content, filter media with RPP showed a much lower breakage rate than the RPP-free blank group. On the other hand, the polymer properly adjusted slurry viscosity, restrained foam floating and collapse, and improved pore-size uniformity. BET test results indicated that filter media prepared under optimal conditions had a specific surface area of 20.151 m^2^/g, and its pores were mostly mesopores of 5–8 nm. This pore-size range allowed phosphate ions to diffuse inside and offered sufficient sites for calcium precipitation reactions. This is the key structural reason why the material achieved better phosphorus-removal performance than ordinary sintered ceramsite.

### 4.3. Mechanism of Coupled Adsorption–Precipitation for Phosphate Removal by Porous PG-Based Filter Media

Combined XRD, SEM and FTIR tests verify that the phosphorus removal of filter media is not a single physical adsorption process. It is a synergistic mechanism of mesoporous physical enrichment and calcium ion chemical precipitation, in which chemical precipitation plays a leading role. During hydration, the PG-based matrix slowly and continuously releases free Ca^2+^ into water, which combines with PO_4_^3−^ to form insoluble calcium phosphate (Ca_3_(PO_4_)_2_) crystals. After adsorption, XRD patterns show characteristic diffraction peaks of calcium phosphate, and SEM results show a large number of needle-like and granular phosphate precipitates filling the pores. FTIR spectra present no obvious shift of Si-O-Si and hydroxyl skeleton absorption peaks, proving that the inorganic matrix has excellent chemical stability. No skeleton corrosion occurs during adsorption, which is conducive to the long-term cyclic application of the filter media.

Conventional sintered ceramsite has limited adsorption capacity, with only 50.28% phosphate-removal efficiency after 6 h, far below the 71.81% obtained for the prepared filter media. The main difference lies in the continuous release of active calcium ions from the PG-based matrix, which supports sustained chemical phosphate removal. By comparison, Gan et al. [16] reported roughly 90% phosphate-removal efficiency for sintered ceramsite prepared at 900 °C using a PG-to-FA mass ratio of 4:2. Without high-temperature calcination, the prepared material has much lower preparation costs. Even though its current phosphate-removal performance is somewhat lower, low-cost surface modification can be applied to enhance its performance for different water-treatment scenarios [34].

### 4.4. Environmental Safety, Research Limitations and Engineering-Application Prospects of PG-Based Filter Media

#### 4.4.1. Environmental Safety and Application Scenarios

Leaching tests under the strong-acid condition specified by GB 5085.3-2007 demonstrate that heavy-metal leaching concentrations of the prepared PG-based filter media are far below the standard limits. Trace heavy-metal impurities contained in PG can be synergistically immobilized by hydrated C-S-H gels and polymer films, which effectively suppress heavy-metal ion release. Nevertheless, under strong-acid conditions, fluoride leaching concentration is close to the threshold values for Class III surface water (GB 3838-2002) and Class III groundwater (GB/T 14848-2017).

This filter material presents application potential for low-cost phosphorus removal from high-phosphorus-containing wastewater, as well as for constructed wetlands and rainwater-purification filtration systems. According to the measured leaching data, the dissolution risk of phosphorus and fluoride remains relatively low under neutral-to-alkaline surface-water and wastewater conditions, while exposure to acidic wastewater environments may trigger certain risks of phosphorus and fluoride release. If the filter media is adopted for in situ remediation projects of surface water and groundwater, long-term rainfall erosion, acid rain infiltration and drying–wetting cycles will continuously promote the dissolution and release of soluble fluoride and phosphorus fractions. Therefore, direct application in sensitive aquatic environments without protective measures is not recommended. Only short-term batch leaching tests were performed in this work; long-term dynamic leaching and soil column percolation tests are still required to further evaluate the cumulative release risk of pollutants. For engineering implementation, surface anti-seepage isolation layers can be equipped, or raw PG can be pre-treated to remove soluble impurities so as to reduce the long-term release risk of fluoride and phosphorus. Considering the leaching characteristics of the present PG-based filter media, further research on modification and stabilization will be carried out in follow-up work to mitigate phosphorus-fluoride leaching risks. Future work will also include the control of leaching pollution risks of the filter media, together with surface modification to enhance nitrogen removal, COD reduction and microbial attachment properties. Further performance verification using real complex wastewater matrices is still required before full-scale engineering application.

#### 4.4.2. Limitations of This Study

This study still has several limitations. First, only static batch adsorption tests are carried out in this work, while dynamic continuous water treatment in filter columns is not simulated. The dynamic adsorption capacity, breakthrough curve and reusability over repeated batch runs, as well as long-term cyclic regeneration performance, need further verification. Second, the tests are only conducted under room temperature and neutral water conditions. The interference of complex actual wastewater environments, such as acid–base conditions, high salinity and coexisting contaminants and natural organic matter, on phosphorus removal efficiency remains unclear. Third, long-term dry–wet cycle and immersion aging tests are not performed, and the attenuation laws of pore structure and mechanical strength of the filter media still require further exploration. Fourth, the phosphate-removal performance achieved in this laboratory batch test is limited, and its removal efficiency for other water-quality indicators, including nitrogen and COD, needs further verification. Fifth, the reusability of the PG-based filter media has not been systematically quantified. Although the filter retains favorable mechanical integrity and permits reuse after simple regeneration, the adsorption capacity can only be partially recovered, owing to the calcium-dominated chemical precipitation mechanism, and the phosphorus uptake capacity gradually decreases with increasing cycle numbers. Cyclic adsorption–desorption experiments to evaluate regeneration efficiency and service life will be performed in future research. After phosphorus adsorption, the spent filter media could be considered for land reclamation and vegetation restoration, only if its pollutant leaching risk satisfies environmental regulatory requirements.

#### 4.4.3. Engineering Application Prospects

Future research can be carried out in the following aspects. (i) Dynamic column tests will be conducted to explore the effects of hydraulic retention time and filter layer thickness on continuous phosphorus removal, and corresponding regeneration processes will be developed. (ii) Optimize polymer dosage to balance material wear resistance and preparation cost. Meanwhile, integrate foaming and molding procedures to establish a continuous preparation process so as to promote pilot-scale and large-scale production. (iii) Further modification studies on specific surface area enhancement will be performed to improve water treatment performance. Targeted modification for different types of wastewater can also be optimized to expand its application scenarios. In general, the polymer-modified high-dosage PG filter media realizes the resource disposal of industrial solid waste and synchronous control of water eutrophication. With the advantages of low cost, environmental friendliness and high performance, the material possesses promising potential for industrial application.

Collectively, this work addresses several key bottlenecks of high-PG systems, including matrix brittleness, the lack of sinter-free porous filter-media preparation routes, and the separation between building-material and water-treatment applications. Through RPP polymer modification and controllable foaming under alkali activation, we achieved dual-functional materials covering a high-strength PG-based cementitious matrix and porous water-treatment filter media for phosphate removal from water.

## 5. Conclusions

This study addresses the difficult disposal of massively accumulated PG solid waste and the high-energy drawback of conventional sintered water-treatment filter media. An alkali-activated PG-based composite cementitious system was prepared with the following optimized formulation: 50 wt.% PG, 35 wt.% cement and 15 wt.% fly ash. Under this optimal matrix formula (water–cement ratio of 0.4, 6 wt.% alkali-activator and 1 wt.% RPP), the PG-based cementitious matrix delivers a 28 d compressive strength of 36.4 MPa and a flexural strength of 8.9 MPa, satisfying the target compressive strength requirement for broad building-material application. RPP was added to relieve the inherent brittleness of the PG matrix, and animal-protein foaming was adopted to build continuous mesoporous structures.

Under optimal preparation conditions, the filter material shows a bulk density of 0.809 g/cm^3^, 1 h water absorption of 39%, and a sum of breakage and wear rates of 4.89%. When tested with simulated phosphate-containing wastewater at an initial concentration of 40 mg/L, the material achieves 71.81% phosphate removal within 6 h, exceeding that of commercial sintered ceramsite (50.28%). Multi-scale characterization results show that phosphate removal is mainly controlled by calcium-induced chemical precipitation, and mesoporous physical adsorption plays an auxiliary role. Leaching toxicity tests confirm that the prepared composite exhibits a low release of harmful ions and meets environmental safety requirements.

The major outcomes obtained in this work are that RPP polymer modification achieves pore-structure regulation and improved toughness of PG-based porous cementitious materials, and that this work identifies the synergistic phosphate-removal mechanism of PG-based porous filter media involving calcium-driven chemical precipitation and mesoporous physical adsorption, which helps advance resource reuse of massively stock-piled PG solid waste. In follow-up work, polymer dosage will be adjusted to balance mechanical performance and preparation cost, and further tests will be carried out to evaluate its practical performance under real-wastewater conditions.

## Figures and Tables

**Table 1:** Main chemical composition of raw materials (wt.%).

Component	CaO	SiO_2_	Al_2_O_3_	Fe_2_O_3_	K_2_O	Na_2_O	MgO	SO_3_	SrO	TiO_2_
PG	39.463	3.587	1.62	1.552	0.358	0.318	0.392	48.667	1.137	0.148
FA	8.082	37.568	32.532	11.287	2.915	1.108	2.106	1.359	0.231	1.628
OPC	57.228	24.876	9.868	4.005	0.963	0.123	0.895	1.188	0.069	0.617

**Table 2:** Details of experimental instruments.

Equipment	Model	Manufacturer	Test Purpose
Electronic balance	AX224 ZH	Ohaus Corporation, Parsippany, NJ, USA	Raw material weighing
Horizontal ball mill	BS-216 B	Aomeng Electric , Yueqing, Zhejiang, China	PG grinding
Electric mixer	R6201 B	Burley Instrument Co., Ltd., Zhejiang, China	Slurry mixing
Foam generator	Customized	Hanjian Energy Saving Technology Co., Ltd., Weihai, China	Foam preparation
Standard curing chamber	YH-30B	Jiguang Test Instrument Co., Ltd., Cangzhou, China	Specimen hydration curing
Automatic flexural & compressive tester	YAW-300 D	Rongna Instrument Equipment Co., Ltd., Shaoxing, Zhejiang, China	Mechanical strength test
Vicat apparatus	Customized	Chuangxin Instrument Co., Ltd., Shangyu, Zhejiang, China	Setting time measurement
UV-Vis spectrophotometer	T6	Persee General Instrument Co., Ltd., Beijing, China	TP concentration detection
Constant temperature shaking bath	XMTD-8222	Jinghong Experimental Equipment Co., Ltd., Shanghai, China	Static phosphorus adsorption test
Vacuum drying oven	DHG-9033 BS-III	Xinmiao Medical Equipment Manufacturing Co., Ltd., Shanghai, China	Sample drying
Leaching device	DSX-280B	Shenan Medical Instrument Co., Ltd., Shanghai, China	Toxicity leaching test
SEM	SU8010	Hitachi Ltd., Tokyo, Japan	Micro-morphology observation
XRD	D2 PHASER	Bruker Corporation, Karlsruhe, Germany	Crystal phase analysis
FT-IR	Nicolet iS5	Thermo Fisher Scientific, Waltham, MA, USA	Functional group characterization
Nitrogen adsorption–desorption apparatus	TriStar 3020	Micromeritics Instrument Corporation, Norcross, GA, USA	Specific surface area and pore structure characterization
XRF	Axios	Panalytical B.V., Almelo, Netherlands	Element composition test

**Table 3:** Effects of water–binder ratio on the setting time of materials.

Water–Binder Ratio	Initial Setting Time (h)	Final Setting Time (h)
0.2	1.62	7.20
0.3	1.35	5.95
0.4	1.43	6.87
0.5	1.77	7.35
0.6	2.36	8.12

**Table 4:** Effects of FA replacement of OPC on the setting time of materials.

FA Replacement (%)	Initial Setting Time (h)	Final Setting Time (h)
0	1.43	6.87
5	1.75	7.18
10	1.99	7.35
15	2.29	7.47
20	2.77	8.14
25	3.52	9.33
30	3.92	9.63

**Table 5:** Effects of alkali-activator dosage on the setting time of materials.

Alkali-Activator Dosage (%)	Initial Setting Time (h)	Final Setting Time (h)
0	3.64	6.96
2	2.45	6.54
4	2.29	6.03
6	2.19	5.93
8	1.77	6.24
10	1.82	6.33

**Table 6:** Effects of RPP dosage on the setting time of materials.

RPP Dosage (%)	Initial Setting Time (h)	Final Setting Time (h)
0	1.34	5.31
0.5	1.78	5.71
1	2.19	5.93
1.5	2.33	6.22
2	2.48	6.57
2.5	2.67	6.94

**Table 7:** Raw-material pollutant fractions, leaching performance and leaching rates of PG-based filter media and relevant standard limits.

Group	Item	Cu	Pb	Cr	Zn	Ba	F	P
Mass fraction in raw materials (wt.%)	PG	0.02	0.023	0.046	0.076	0.229	0.16	1.187
	FA	0.037	0.019	0.025	-	-	-	0.231
	OPC	-	-	-	-	-	-	0.029
Leaching concentration (mg/L)	Raw PG	0.003	-	0.079	0.012	0.117	13.319	32.107
	Filter media	n.d. <5 × 10^−4^	0.0007	0.016	n.d. <1.8 × 10^−3^	0.087	1.027	0.253
Regulatory limits (mg/L)	GB 5085.3-2007	100	5	15	100	100	100	-
	GB/T 14848-2017 (Class III groundwater)	1	0.01	0.05 *	1	0.7	1	-
	GB 3838-2002 (Class III surface water)	1	0.05	0.05 *	1		1	0.2; 0.05 (lakes/reservoirs)
Leaching rate (%)	Filter media	-	0.0049	0.0598	-	0.0781	1.284	0.04

Note: * Cr threshold calculated as Cr (VI). The total pollutant content for leaching-rate calculation is a theoretical weighted value derived from raw-material elemental data. “-” denotes not detected or unavailable. GB 5085.3-2007: Identification standards for hazardous wastes—Identification for extraction toxicity; GB/T 14848-2017: Groundwater quality standard (Class III); GB 3838-2002: Environmental quality standards for surface water (Class III).

**Table 8:** Analysis of variance for phosphorus-removal performance.

Group 1	Group 2	Mean Difference	Standard Error	F-Value	95% Confidence Interval	*p*-Value
PG-based filter media	Sintered ceramic particles	0.7207	0.16	20.29	(0.3955, 1.0458)	<0.0001 (Significant)

**Table 9:** Kinetic parameters for phosphate adsorption onto PG-based filter media.

Sample	Pseudo-First-Order Model			Pseudo-Second-Order Model		
	*q*_e,cal_/*q*_e,exp_ (mg/g)	*k*_1_	*R* ^2^	*q*_e,cal_/*q*_e,exp_ (mg/g)	*k*_1_	*R* ^2^
PG-based filter media	0.59/0.57	0.53	0.987	0.76/0.57	0.69	0.995

Note: *q*_e,cal_-calculated equilibrium adsorption capacity; *q*_e,exp_-experimental equilibrium adsorption capacity. *k*_1_ and *k*_2_ are rate constants of pseudo-first-order and pseudo-second-order models, respectively.

**Table 10:** Main chemical components of PG-based filter media (wt.%).

Al_2_O_3_	SiO_2_	SO_3_	CaO	Fe_2_O_3_	MgO	TiO_2_	Others
5.63	11.92	37.56	40.84	2.46	0.23	0.42	0.87

## Data Availability

The raw data supporting the conclusions of this article will be made available by the authors on request. No data access restrictions apply.
